# Supplementary figures and images for: Neuropeptide-mediated synaptic plasticity regulates context-dependent mating behaviors in Drosophila
Source: PLoS Biol. 2025 Sep 4;23(9):e3003330. doi: 10.1371/journal.pbio.3003330 (PMC12410882; doi:10.1371/journal.pbio.3003330)

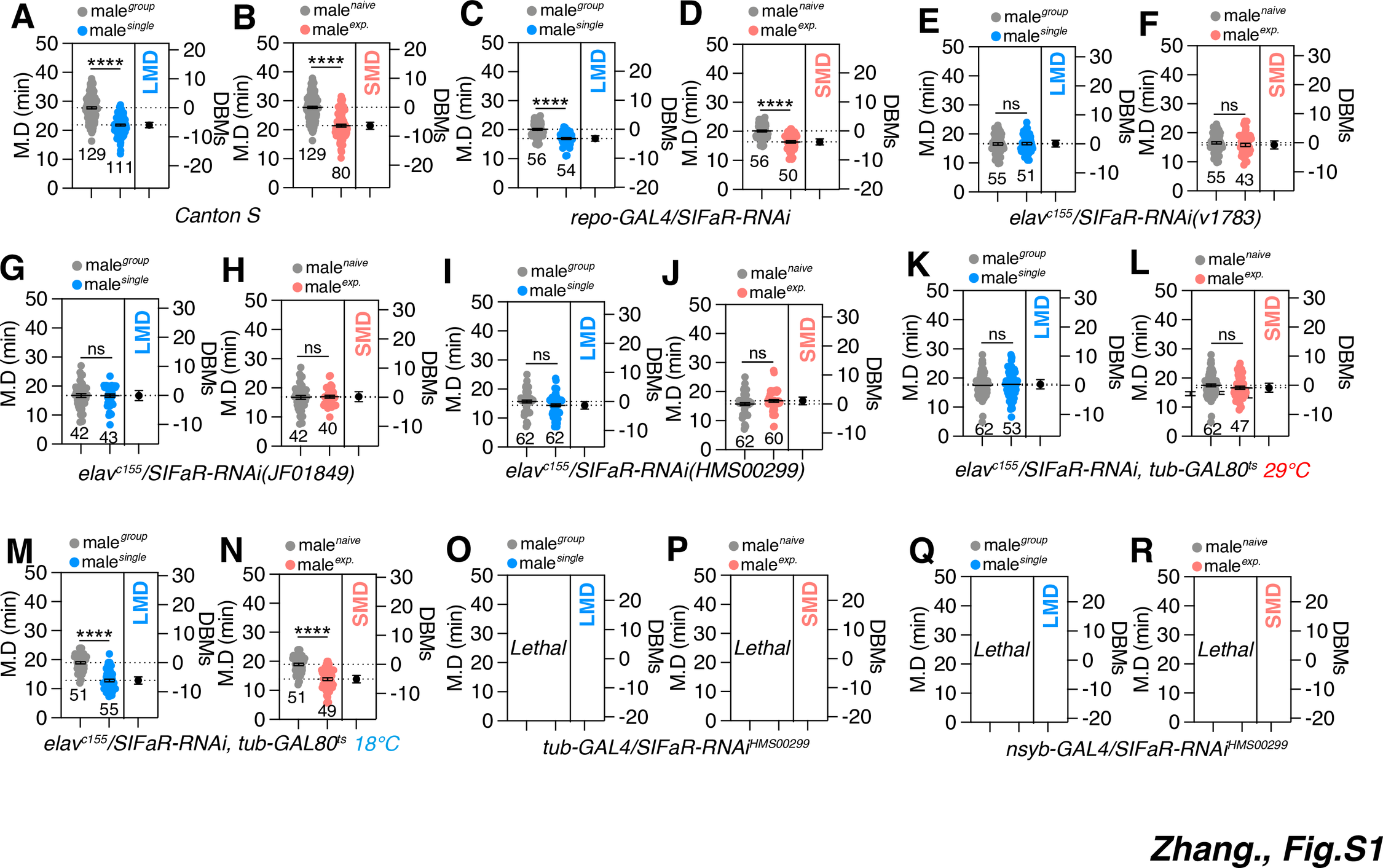

Supplement: S1 Fig — (A, B) LMD and SMD assays of Conton-S (WT) (two-tailed unpaired t test). In all plots and statistical tests. Data are presented as mean ± s.e.m. ns = not significant (p > 0.05), *p < 0.05, **p < 0.01, ***p < 0.001, ****p < 0.0001. Sample sizes (n) are indicated in the figure panels. (C, D) LMD and SMD assays for repo-GAL4 mediated knockdown of SIFaR via SIFaR-RNAi (two-tailed unpaired t test). In all plots and statistical tests. Data are presented as mean ± s.e.m. ns = not significant (p > 0.05), *p < 0.05, **p < 0.01, ***p < 0.001, ****p < 0.0001. Sample sizes (n) are indicated in the figure panels. (E–J) LMD and SMD assays for elavc155-mediated knockdown of SIFaR via (E, F) SIFaR-RNAi (JF01849), (G, H) SIFaR-RNAi (HMS00299), and (I, J) SIFaR-RNAi (HMS00299) (two-tailed unpaired t test). In all plots and statistical tests. Data are presented as mean ± s.e.m. ns = not significant (p > 0.05), *p < 0.05, **p < 0.01, ***p < 0.001, ****p < 0.0001. Sample sizes (n) are indicated in the figure panels. (K, L) LMD and SMD assays for elavc155-mediated knockdown of SIFaR via SIFaR-RNAi together with tub-GAL80ts in 29 °C (two-tailed unpaired t test). In all plots and statistical tests. Data are presented as mean ± s.e.m. ns = not significant (p > 0.05), *p < 0.05, **p < 0.01, ***p < 0.001, ****p < 0.0001. Sample sizes (n) are indicated in the figure panels. (M, N) LMD and SMD assays for elavc155-mediated knockdown of SIFaR via SIFaR-RNAi together with tub-GAL80ts in 18 °C (two-tailed unpaired t test). In all plots and statistical tests. Data are presented as mean ± s.e.m. ns = not significant (p > 0.05), *p < 0.05, **p < 0.01, ***p < 0.001, ****p < 0.0001. Sample sizes (n) are indicated in the figure panels. (O–R) Drosophila lethality induced by nsyb-GAL4 and tub-GAL4 knockdown of SIFaR via SIFaR-RNAi. Underlying data for all graphs can be found in file S1 Data. (TIF) [file pbio.3003330.s001.tif]

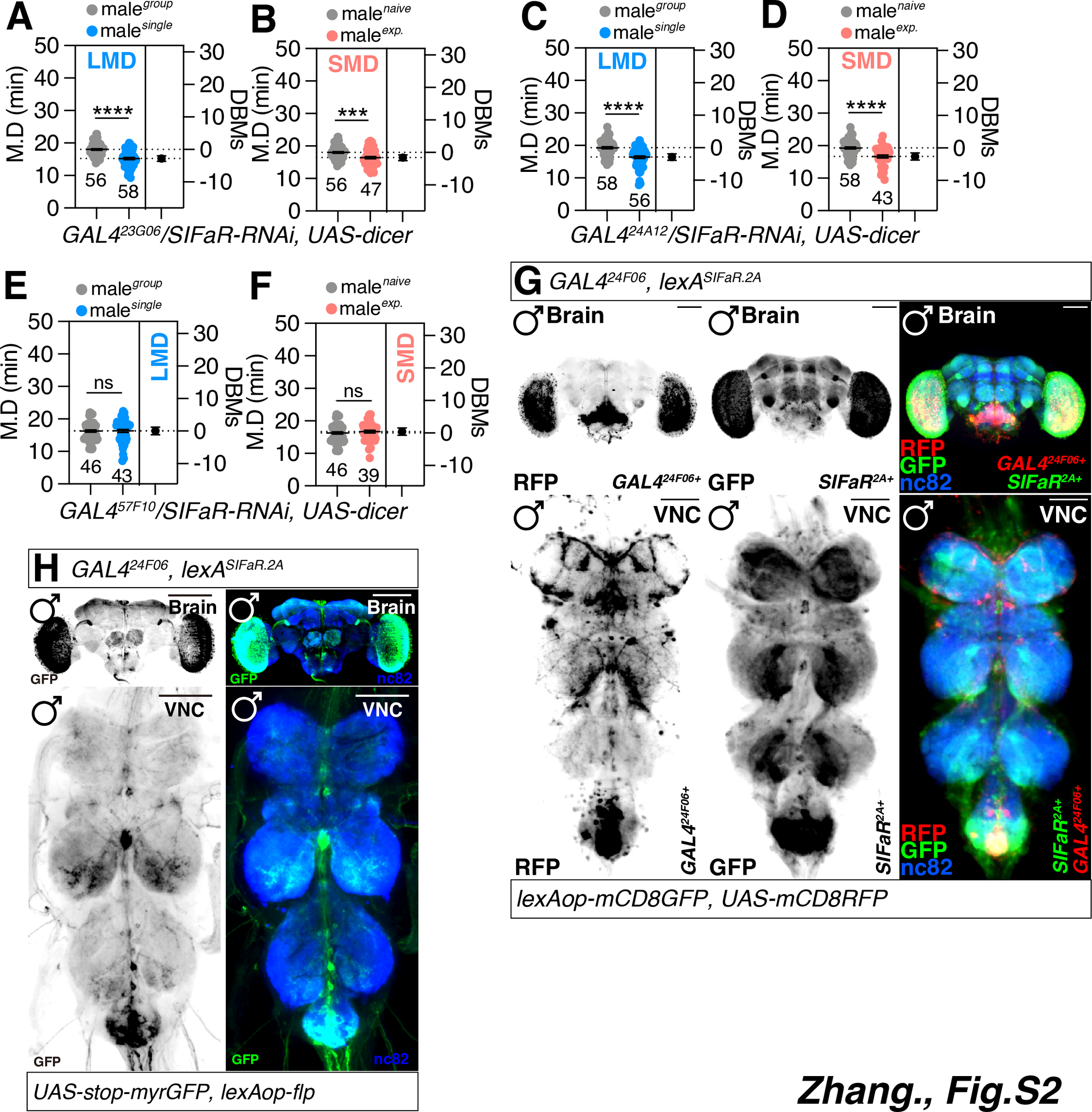

Supplement: S2 Fig — (A–F) LMD and SMD assays of (A, B) GAL423G06, (C, D) GAL424A12, and (E, F) GAL457F10 mediated knockdown of SIFaR via SIFaR-RNAi together with UAS-dicer (two-tailed unpaired t test). In all plots and statistical tests. Data are presented as mean ± s.e.m. ns = not significant (p > 0.05), *p < 0.05, **p < 0.01, ***p < 0.001, ****p < 0.0001. Sample sizes (n) are indicated in the figure panels. (G) Male flies brain and VNC expressing lexASIFaR.2A and GAL424F06 drivers together with UAS-mCD8RFP and lexAop-mCD8GFP were immunostained with anti-GFP (green), anti-DsRed (red), and anti-nc82 (blue) antibodies. Scale bars represent 100 μm. Boxes indicate the magnified regions of interest presented in the bottom panels. The panels presented as gray scale are to clearly show the axon projection patterns of neurons in brain and VNC labeled by lexASIFaR.2A and GAL424F06 driver. (H) Male flies brain and VNC expressing lexASIFaR.2A and GAL424F06 drivers together with UAS-stop-myrGFP and lexAop-flp were immunostained with anti-GFP (green) and anti-nc82 (blue). Underlying data for all graphs can be found in file S1 Data. (TIF) [file pbio.3003330.s002.tif]

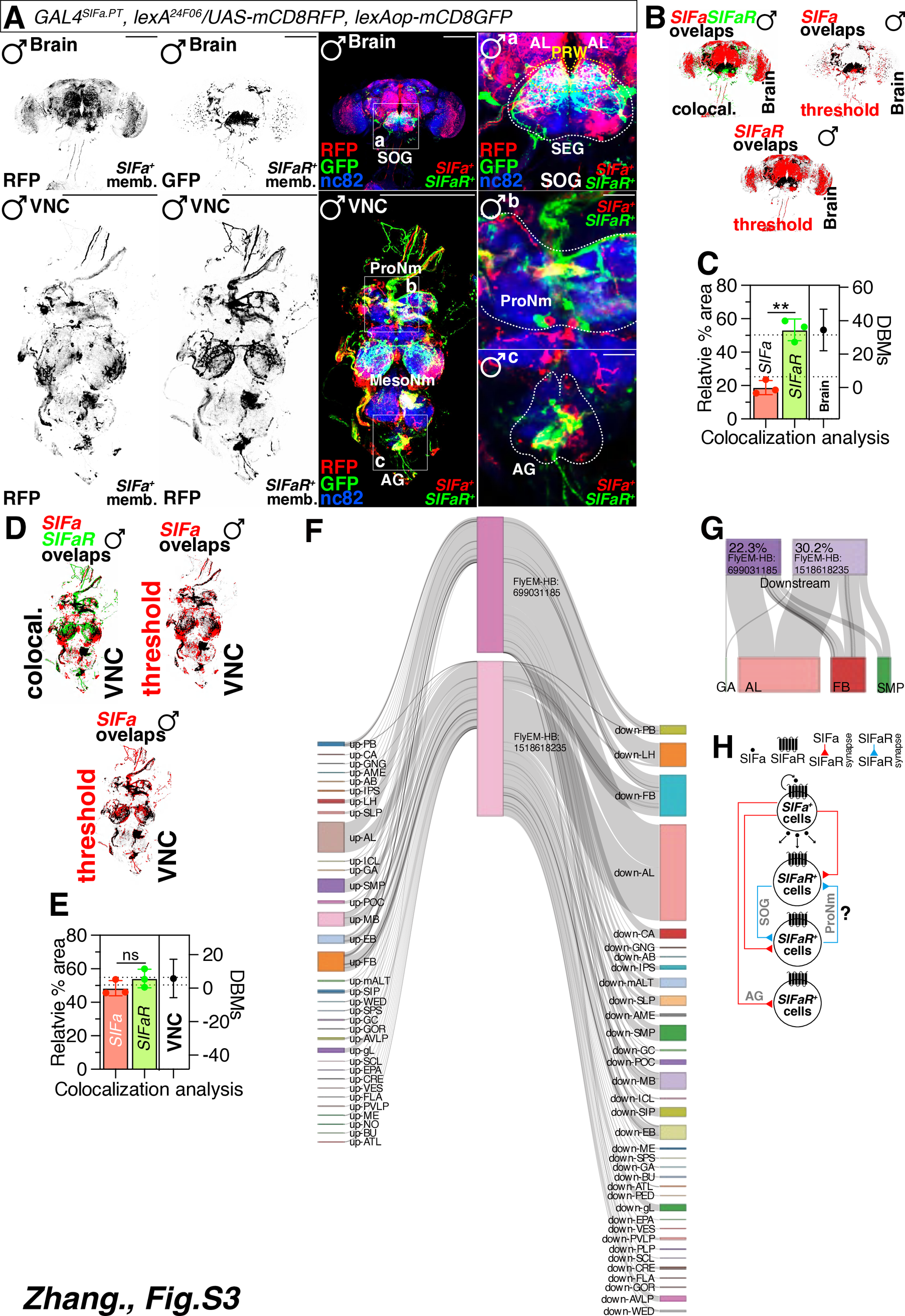

Supplement: S3 Fig — (A) Male flies brain expressing GAL4SIFa.PT and lexA24F06 drivers together with UAS-mCD8RFP and lexAop-mCD8GFP were immunostained with anti-GFP (green), anti-DsRed (red), and anti-nc82 (blue) antibodies. Boxes and dashed circles indicate the magnified regions of interest presented in the bottom panels. The left two panels are presented as a gray scale to clearly show the axon projection patterns of neurons labeled by GAL4SIFa.PT and lexA24F06 driver. Scale bars represent 100 μm in brain and VNC panels, and 25 μm in other panels. (B–E) Colocalization analysis of GFP and RFP staining, normalized to total GFP and RFP areas. Bars represent the mean GFP (green column) and RFP (red column) fluorescence level with error bars representing SEM. DBMs represent the difference between means. The regions analyzed are clearly marked on the figure. Asterisks represent significant differences, as revealed by the Student t test and ns represents non-significant difference (*p < 0.05, **p < 0.01, ***p < 0.001, ****p < 0.0001). The same symbols for statistical significance are used in all other figures. See the Materials and methods for a detailed description of the colocalization analysis used in this study. (F) Sankey diagram illustrating the connectivity of two SIFa neurons (PDM34) across brain right regions. The diagram visualizes synaptic connectivity data obtained from Virtual Fly Brain: “Connectivity per region for SIFa (FlyEM-HB:1418618235)” and “Connectivity per region for SIFa (PDM34) (FlyEM-HB:699031185).” Upstream regions (labeled “up-”) represent brain areas projecting signals to SIFa neurons, while downstream regions (labeled “down-”) represent brain areas receiving signals from these neurons. The thickness of each link corresponds to the number of synaptic connections. See the Materials and methods for a detailed description of the connectome analysis used in this study. (G) Sankey diagram illustrating the downstream connections of two SIFa neurons located in the right [file pbio.3003330.s003.tif]

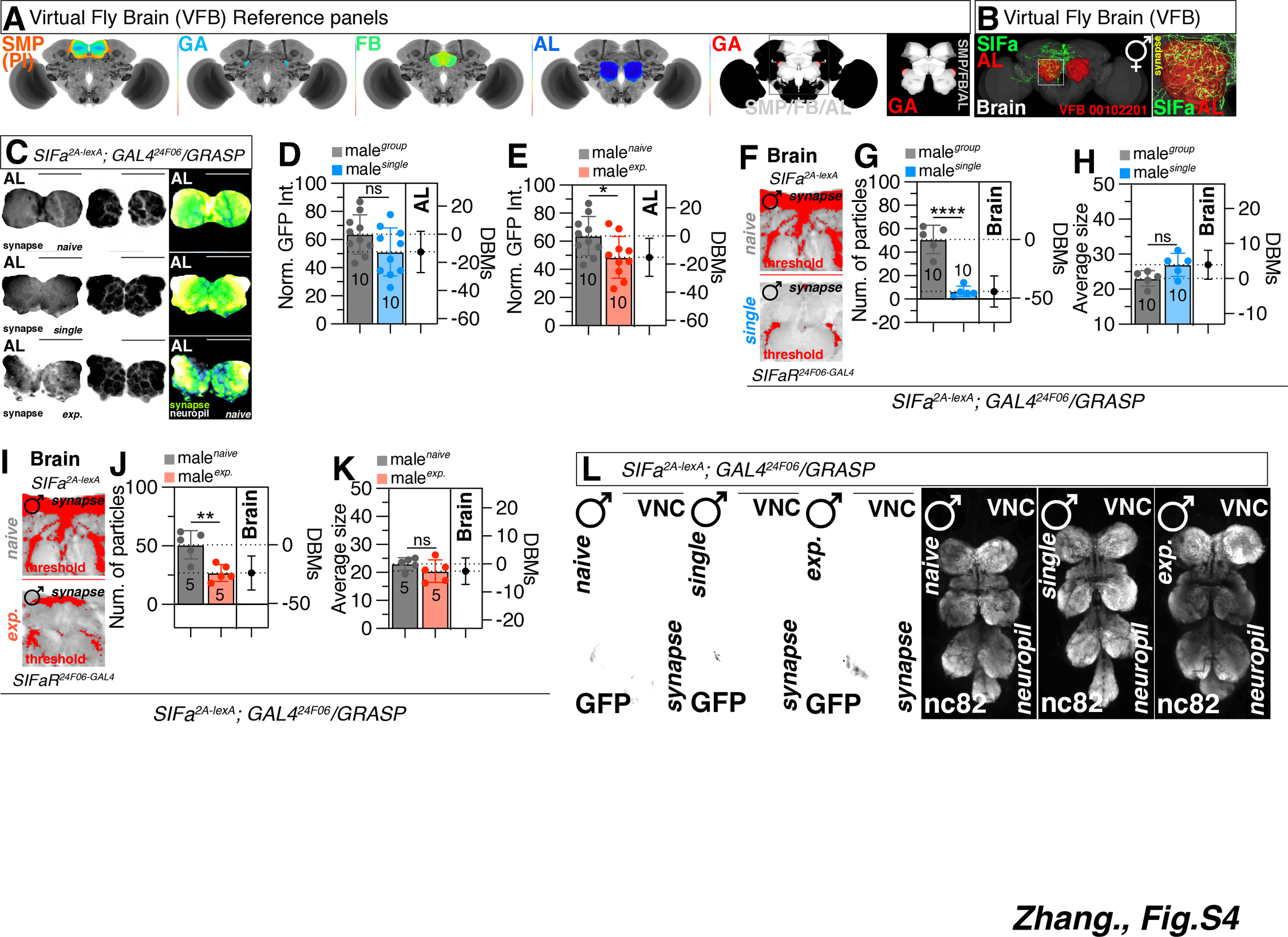

Supplement: S4 Fig — (A) Virtual Fly Brain (VFB) visualization of SMP, GA, FB, and AL brain regions in Drosophila. In the last two panels, the gray area represents the overlapping region of SMP/FB/AL, and the red area represents GA. (B) Co-locolization between SIFa and AL in Virtual Fly Brain (VFB). White boxes indicate the magnified regions of interest presented in the right panel. The yellow area represents synaptic connections formed by SIFa (green) and AL (red). (C) GRASP assay for SIFa2A-lexA and GAL424F06 in AL region of naïve (top three columns), single (middle three columns) and experienced (bottom three columns) male flies. Male flies expressing SIFa2A-lexA, GAL424F06 and lexAop-nsyb-spGFP1-10, UAS-CD4-spGFP11 were dissected after 5 days of growth. GFP is pseudo-colored as “Green fire blue”. (D, E) Quantification of relative value for synaptic intensity (two-tailed unpaired t test). In all plots and statistical tests. Data are presented as mean ± s.e.m. ns = not significant (p > 0.05), *p < 0.05, **p < 0.01, ***p < 0.001, ****p < 0.0001. Sample sizes (n) are indicated in the figure panels. (F) The synaptic interactions visualized utilizing the GRASP system in naïve and single male flies. The GFP fluorescence was processed using ImageJ software, where a threshold function was applied to distinguish fluorescence from the background. (G) Quantification of synaptic puncta formed between SIFa2A-lexA and GAL424F06 in brain between naïve and single male flies. The synaptic interactions were visualized utilizing the GRASP system in male flies. Bars represent the mean particle number with error bars representing SEM. Asterisks represent significant differences, as revealed by the Student t test and ns represents non-significant difference (*p < 0.05, **p < 0.01, ***p < 0.001, ****p < 0.0001). Sample sizes (n) are indicated in the figure panels. See the Materials and methods for a detailed description of the particle analysis used in this study. (H) Quantification of average synapse size [file pbio.3003330.s004.tif]

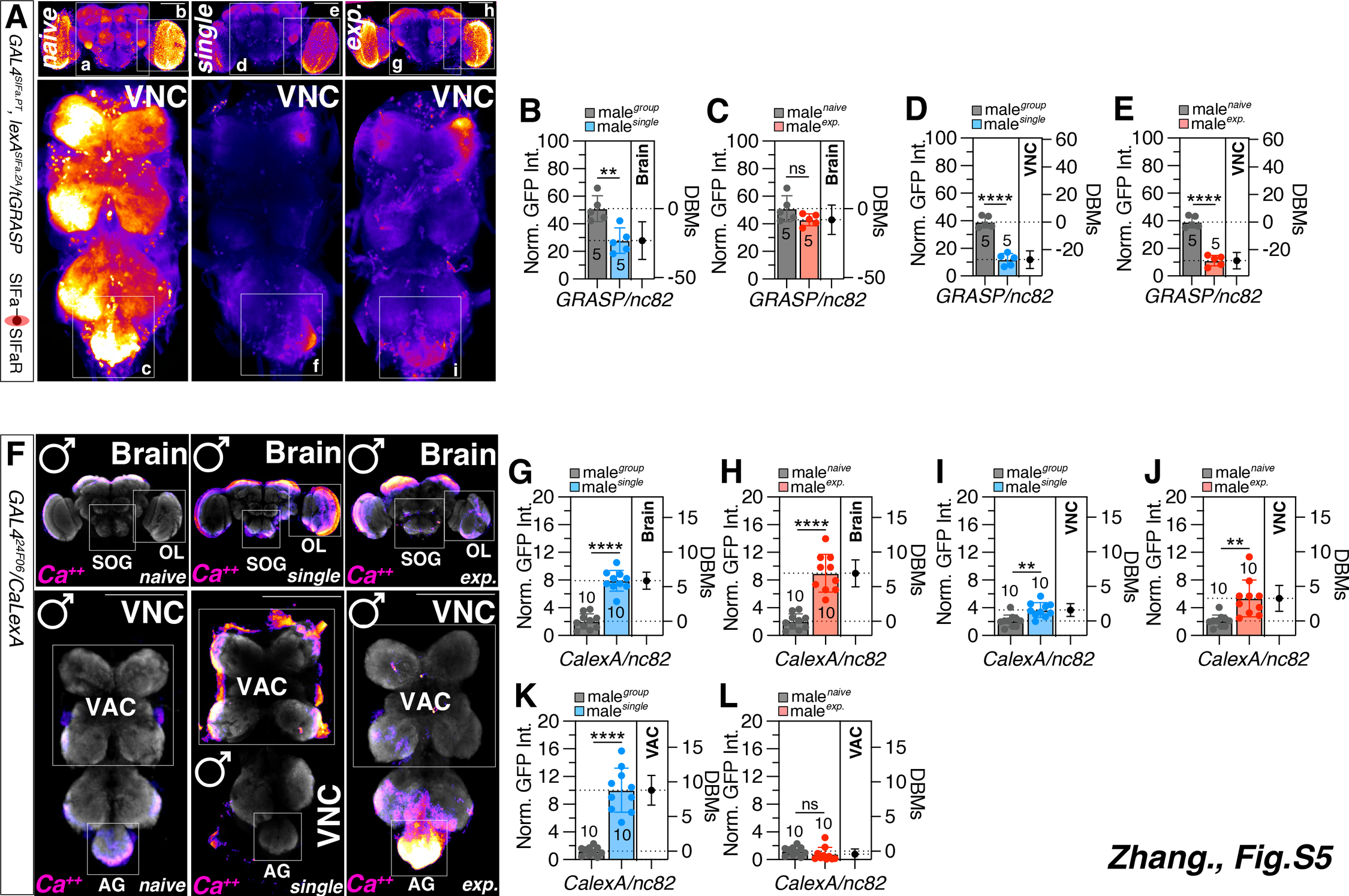

Supplement: S5 Fig — (A–E) Quantification of synaptic relative intensity formed between GAL4SIFa.PT and lexASIFaR.2A in Brain and VNC between (B, D) naïve and single male flies; (C, E) naïve and experienced male flies (two-tailed unpaired t test). In all plots and statistical tests. Data are presented as mean ± s.e.m. ns = not significant (p > 0.05), *p < 0.05, **p < 0.01, ***p < 0.001, ****p < 0.0001. Sample sizes (n) are indicated in the figure panels. The synaptic interactions were visualized utilizing the tGRASP system in naïve, single and experienced male flies. Synaptic transmission occurs from GAL4SIFa.PT to lexASIFaR.2A. (F) Different levels of neural activity of the brain as revealed by the CaLexA system in naïve, single and experienced flies. Male flies expressing GAL424F06 along with LexAop-CD2-GFP, UAS-mLexA-VP16-NFAT and LexAop-CD8-GFP-A2-CD8-GFP were dissected after 5 days of growth (mated male flies had 1-day of sexual experience with virgin females). The dissected brains were then immunostained with anti-GFP (green) and anti-nc82 (blue). GFP is pseudo-colored as “red hot”. Boxes indicate the magnified regions of interest presented in the bottom panels. Scale bars represent 100 μm in brain and VNC panels. (G–L) Quantification of relative intensity value for GFP fluorescence (two-tailed unpaired t test). In all plots and statistical tests. Data are presented as mean ± s.e.m. ns = not significant (p > 0.05), *p < 0.05, **p < 0.01, ***p < 0.001, ****p < 0.0001. Sample sizes (n) are indicated in the figure panels. Underlying data for all graphs can be found in file S1 Data. (TIF) [file pbio.3003330.s005.tif]

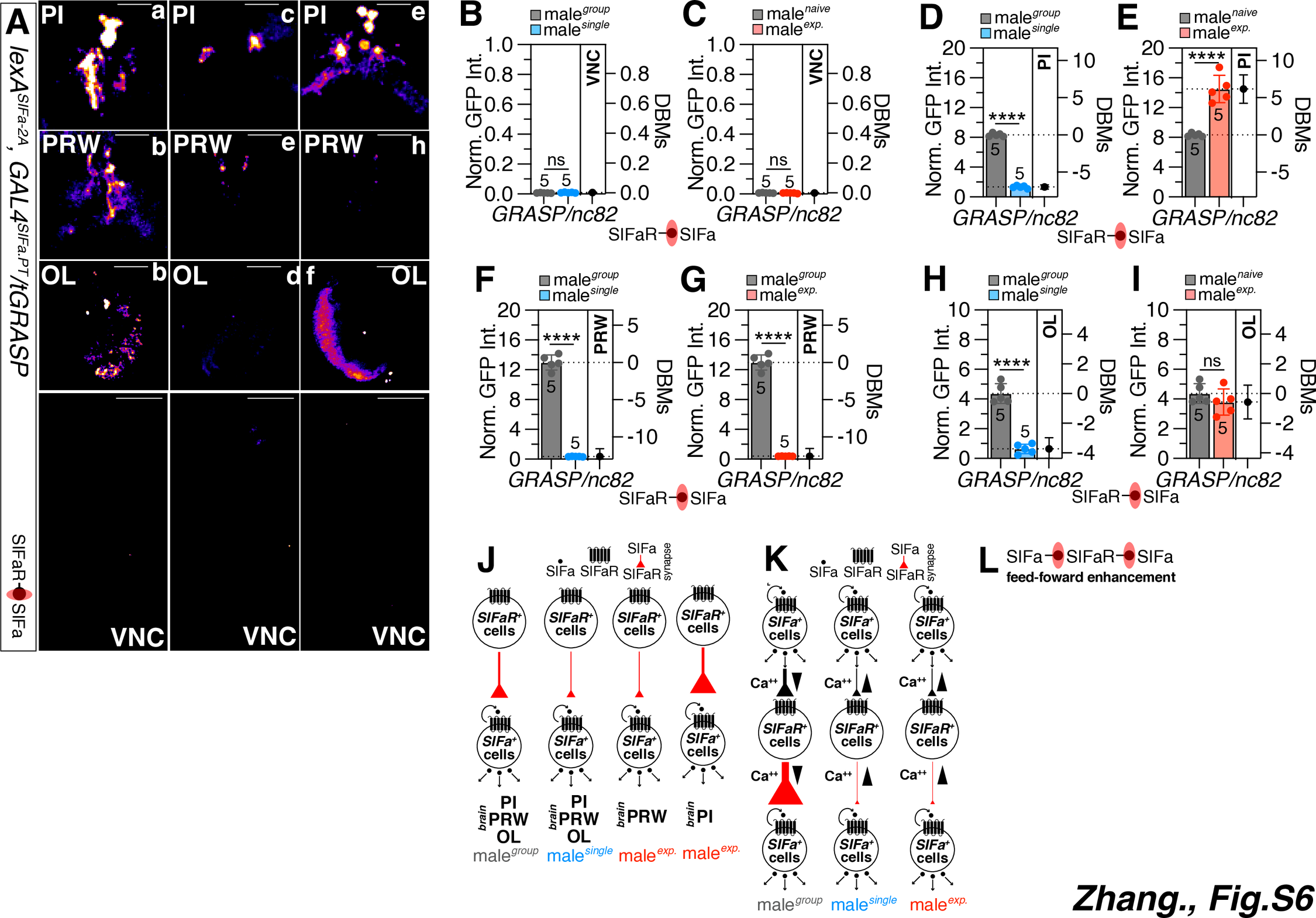

Supplement: S6 Fig — (A) tGRASP assay for GAL4SIFa.PT and lexASIFaR-2A in PI, PRW, OL and VNC region of male flies. Male flies expressing GAL4SIFa.PT and lexASIFaR-2A and LexAop-2-pre-t-GRASP, UAS-post-t-GRASP were dissected after 5 days of growth. Brains of male flies were immunostained with anti-GFP (green) and anti-nc82 (blue) antibodies. GFP is pseudo-colored as “red hot”. Boxes indicate the magnified regions of interest presented in the bottom panels. Scale bars represent 100 μm in VNC panels, 25 μm in PI and PRW panels, and 50 μm in OL panels. Synaptic transmission occurs from lexASIFaR-2A to GAL4SIFa.PT. (B–I) Quantification of synaptic relative intensity formed between GAL4SIFa.PT and lexASIFaR-2A in (J) brain, (L) PI, (N) PRW and (P) OL between naïve and single male flies (two-tailed unpaired t test). In all plots and statistical tests. Data are presented as mean ± s.e.m. ns = not significant (p > 0.05), *p < 0.05, **p < 0.01, ***p < 0.001, ****p < 0.0001. Sample sizes (n) are indicated in the figure panels. The same quantification was performed for the relative synaptic intensity in these regions between naïve and experienced male flies. The synaptic interactions were visualized utilizing the tGRASP system in naïve, single and experienced male flies. (J) Diagram of differential SIFa-SIFaR signaling across various regions of the CNS in male Drosophila melanogaster, contingent upon diverse social contexts. (K) Schematic representation of neuronal circuits modulating CNS internal states through feed-forward augmentation in response to social contexts. (L) Feed-forward enhancement circuits of SIFa-SIFaR-SIFa. Underlying data for all graphs can be found in file S1 Data. (TIF) [file pbio.3003330.s006.tif]

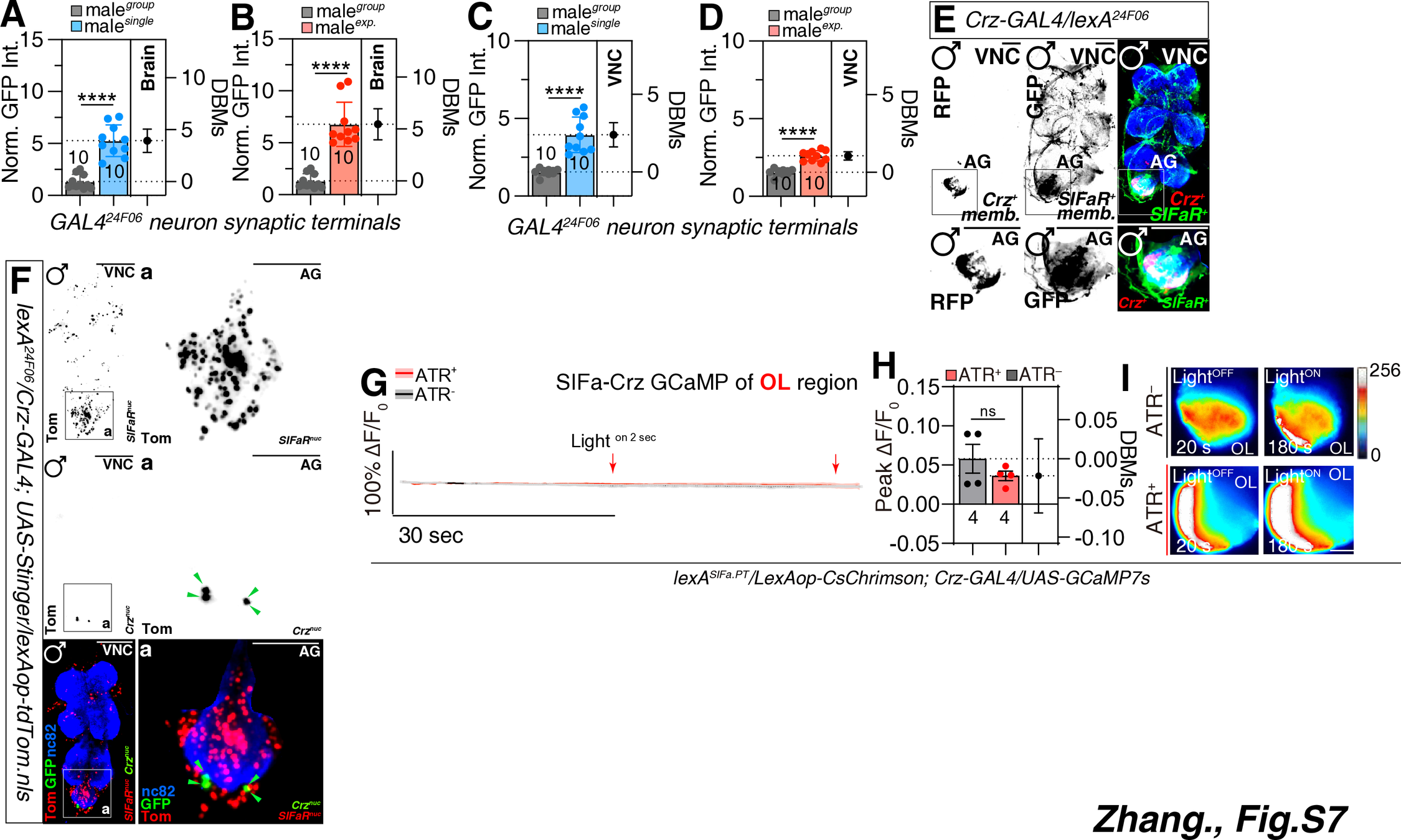

Supplement: S7 Fig — (A–D) Quantification of relative intensity value for GFP fluorescence in brain (A, B), and VNC (C, D) (two-tailed unpaired t test). In all plots and statistical tests. Data are presented as mean ± s.e.m. ns = not significant (p > 0.05), *p < 0.05, **p < 0.01, ***p < 0.001, ****p < 0.0001. Sample sizes (n) are indicated in the figure panels. (E) Male flies VNC expressing Crz-GAL4 and lexA24F06 drivers together with UAS-mCD8RFP and lexAop-mCD8GFP were immunostained with anti-GFP (green), anti-DsRed (red), and anti-nc82 (blue) antibodies. Scale bars represent 100 μm in VNC panels and 50 μm in AG panels. Boxes indicate the magnified regions of interest presented in the bottom panels. The panels presented as a gray scale are to clearly show the nucleus in the adult VNC and AG labeled by Crz-GAL4 and lexA24F06 driver. (F) Male flies VNC expressing Crz-GAL4 and lexA24F06 drivers together with UAS-Stinger and lexAop-tdTomato.nls were immunostained with anti-GFP (green), anti-DsRed (red), and anti-nc82 (blue) antibodies. Scale bars represent 50 μm. Boxes indicate the magnified regions of interest presented in the bottom panels. The upper panels are presented as a gray scale to clearly show the nucleus in the adult VNC and AG labeled by Crz-GAL4 and lexA24F06 driver. Green arrows indicate Crz+ nucleus. (G–I) Crzs slightly respond to SIFas activity in OL. Fluorescence changes (ΔF/F0) of GCaMP7s in Crzs after optogenetic stimulation of SIFas (two-tailed unpaired t test). In all plots and statistical tests. Data are presented as mean ± s.e.m. ns = not significant (p > 0.05), *p < 0.05, **p < 0.01, ***p < 0.001, ****p < 0.0001. Sample sizes (n) are indicated in the figure panels. LED light were fired 2 s after 30 s of dark. N = 4 in each group. Scale bars represent 50 μm. Underlying data for all graphs can be found in file S1 Data. (TIF) [file pbio.3003330.s007.tif]

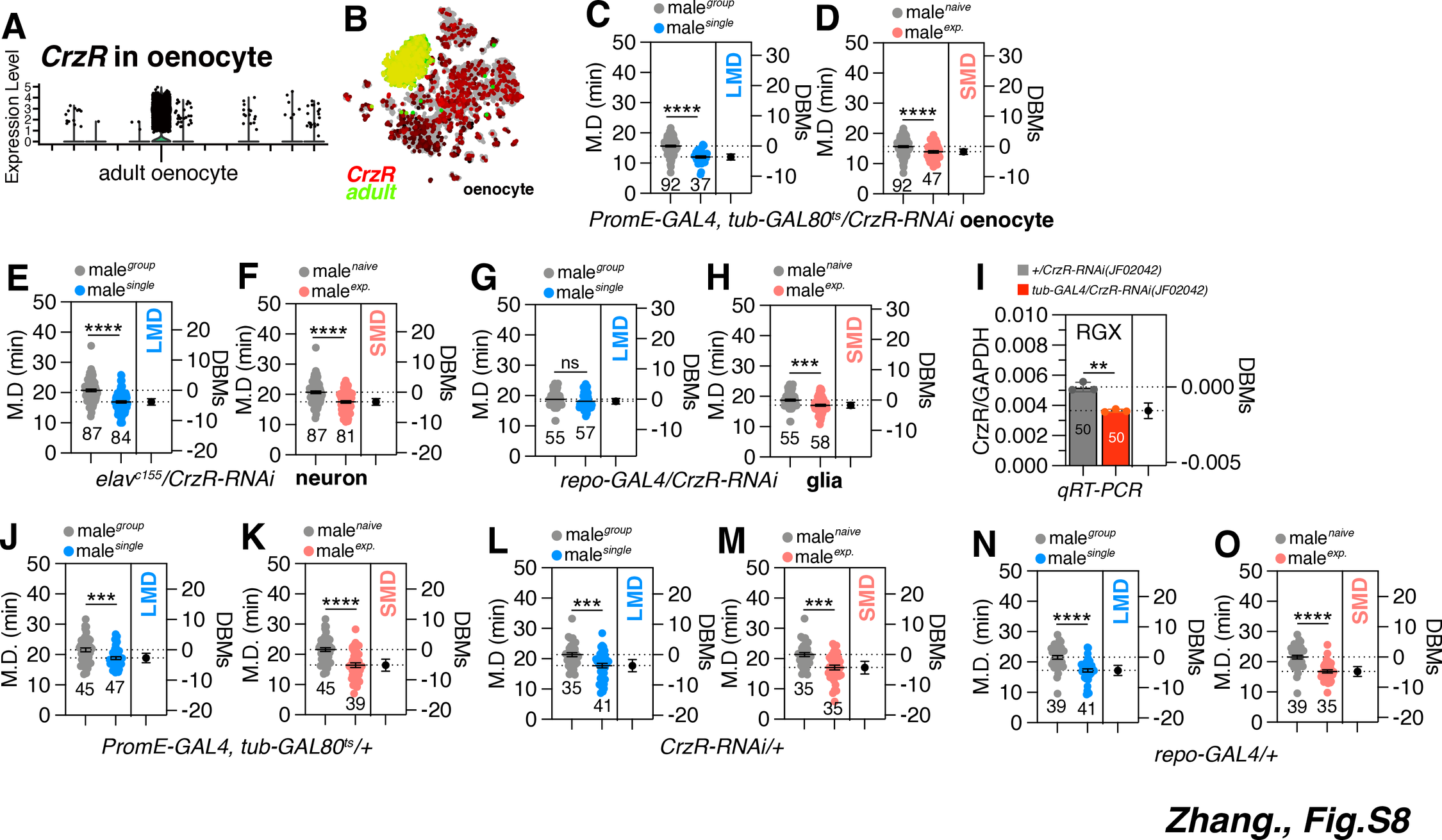

Supplement: S8 Fig — (A) Dot plot depicting the expression levels of CrzR in ‘adult oenocyte’ as annotated by the Fly Cell Atlas (FCA) within the oenocyte tissue. The gene expression levels for individual cells were normalized using the ‘LogNormalize’ method with a scale factor of 10,000, followed by scaling of all genes. See the Materials and methods for a detailed description of the single-nucleus RNA-sequencing analyses used in this study. (B) Each tSNE visualization depicts the coexpression patterns of genes, with each color corresponding to the genes listed on the left, right, and bottom of the plot. The tissue name, as referenced on the Fly SCope website is indicated in the upper left corner of the tSNE plot. See the Materials and methods for a detailed description of the single-nucleus RNA-sequencing analyses used in this study. (C, D) LMD and SMD assays for PromE-GAL4 mediated knockdown of SIFaR via SIFaR-RNAi together with tub-GAL80ts (two-tailed unpaired t test). In all plots and statistical tests. Data are presented as mean ± s.e.m. ns = not significant (p > 0.05), *p < 0.05, **p < 0.01, ***p < 0.001, ****p < 0.0001. Sample sizes (n) are indicated in the figure panels. (E, F) LMD and SMD assays for elavc155 (neuron) -mediated knockdown of CrzR via CrzR-RNAi (two-tailed unpaired t test). In all plots and statistical tests. Data are presented as mean ± s.e.m. ns = not significant (p > 0.05), *p < 0.05, **p < 0.01, ***p < 0.001, ****p < 0.0001. Sample sizes (n) are indicated in the figure panels. (G, H) LMD and SMD assays for repo-GAL4 (glia) -mediated knockdown of CrzR via CrzR-RNAi (two-tailed unpaired t test). In all plots and statistical tests. Data are presented as mean ± s.e.m. ns = not significant (p > 0.05), *p < 0.05, **p < 0.01, ***p < 0.001, ****p < 0.0001. Sample sizes (n) are indicated in the figure panels. (I) The results of qRT-PCR for SIFaR gene expression. The gray bar represents the control group, which has the genotype +/CrzR-RNAi. The red bar indicates the ex [file pbio.3003330.s008.tif]

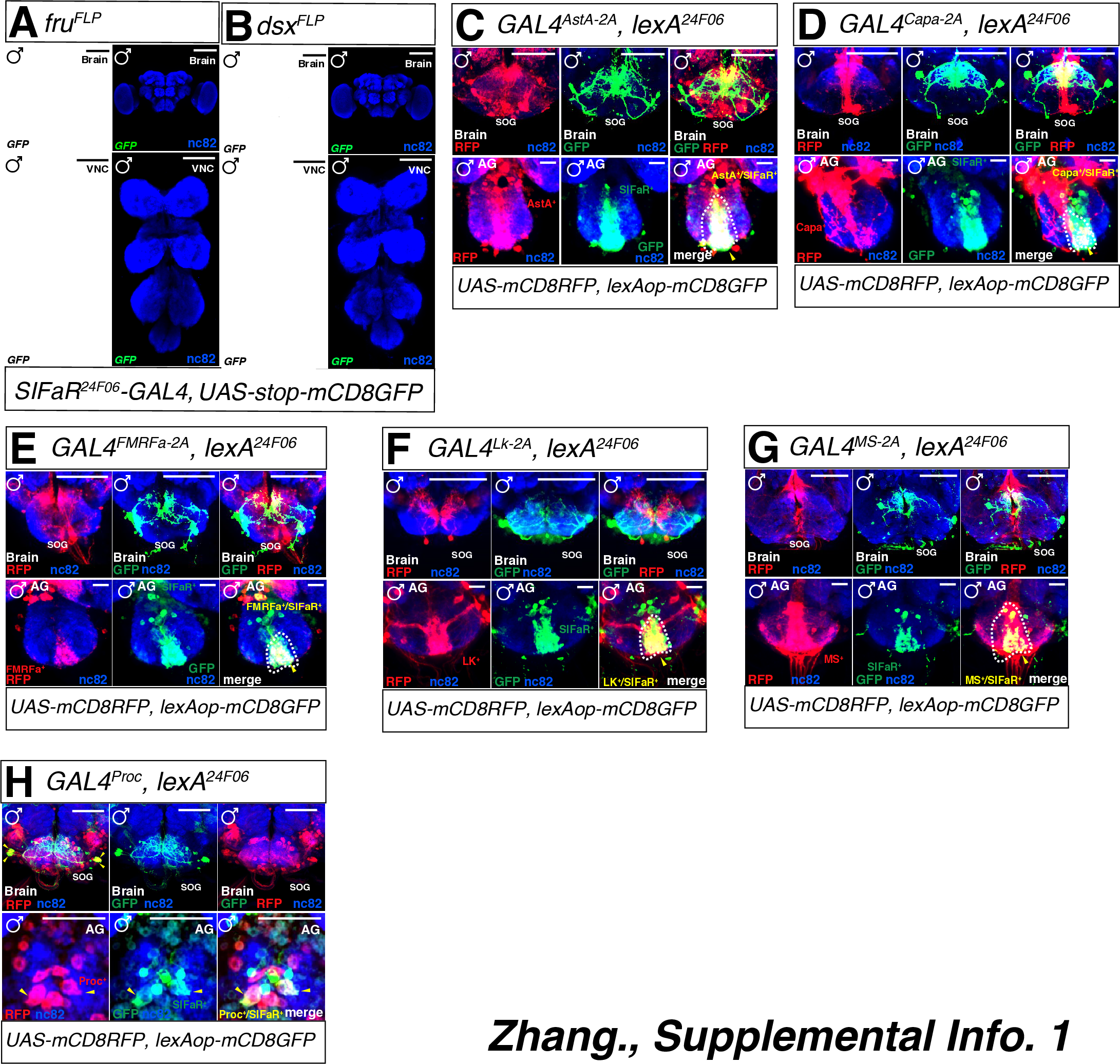

Supplement: S1 Supplemental information — (A) Male flies brain expressing GAL424F06 and fruFLP together with UAS-stop-mCD8GFP were immunostained with anti-GFP (green) and anti-nc82 (blue) antibodies. Scale bars represent 100 μm in brain and 50 μm in VNC panels. (B) Male flies brain expressing GAL424F06 and dsxFLP together with UAS-stop-mCD8GFP were immunostained with anti-GFP (green) and anti-nc82 (blue) antibodies. Scale bars represent 100 μm in brain and 50 μm in VNC panels. (C) Male flies brain expressing GAL4AstA-2A and lexA24F06 drivers together with UAS-mCD8RFP and lexAop-mCD8GFP were immunostained with anti-GFP (green), anti-DsRed (red), and anti-nc82 (blue) antibodies. Dashed circles indicate the regions of interest. Scale bars represent 50 μm in SOG and 20 μm in AG panel. (D) Male flies brain expressing GAL4Capa-2A and lexA24F06 drivers together with UAS-mCD8RFP and lexAop-mCD8GFP were immunostained with anti-GFP (green), anti-DsRed (red), and anti-nc82 (blue) antibodies. Dashed circles indicate the regions of interest. Scale bars represent 50 μm in SOG and 20 μm in AG panel. (E) Male flies brain expressing GAL4FMRFa-2A and lexA24F06 drivers together with UAS-mCD8RFP and lexAop-mCD8GFP were immunostained with anti-GFP (green), anti-DsRed (red), and anti-nc82 (blue) antibodies. Dashed circles indicate the regions of interest. Scale bars represent 50 μm in SOG and 20 μm in AG panel. (F) Male flies brain expressing GAL4Lk-2A and lexA24F06 drivers together with UAS-mCD8RFP and lexAop-mCD8GFP were immunostained with anti-GFP (green), anti-DsRed (red) and anti-nc82 (blue) antibodies. Dashed circles indicate the regions of interest. Scale bars represent 50 μm in SOG and 20 μm in AG panel. (G) Male flies brain expressing GAL4MS-2A and lexA24F06 drivers together with UAS-mCD8RFP and lexAop-mCD8GFP were immunostained with anti-GFP (green), anti-DsRed (red), and anti-nc82 (blue) antibodies. Dashed circles indicate the regions of interest. Scale bars represent 50 μm in SOG and 20 μm in AG panel. (H) Male flies [file pbio.3003330.s011.tif]

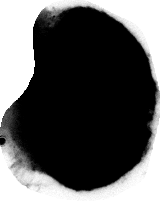

Supplement: S2 Supplemental information — (ZIP) [file pbio.3003330.s012.zip › Quantification-Images/Naive-OL-1-nc82.tif]

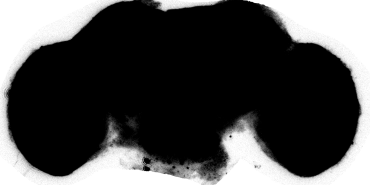

Supplement: S2 Supplemental information — (ZIP) [file pbio.3003330.s012.zip › Quantification-Images/Naive-brain-2-nc82.tif]

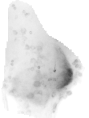

Supplement: S2 Supplemental information — (ZIP) [file pbio.3003330.s012.zip › Quantification-Images/SIngle-AG-1-GFP.tif]

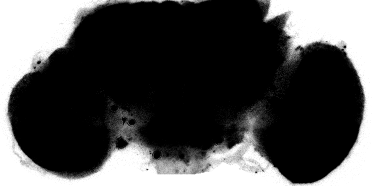

Supplement: S2 Supplemental information — (ZIP) [file pbio.3003330.s012.zip › Quantification-Images/Single-brain-1-nc82.tif]

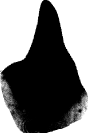

Supplement: S2 Supplemental information — (ZIP) [file pbio.3003330.s012.zip › Quantification-Images/Naive-AG-1-nc82.tif]

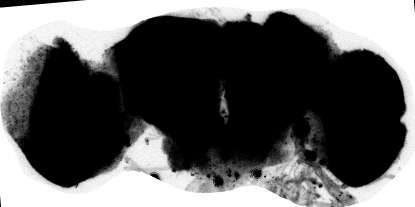

Supplement: S2 Supplemental information — (ZIP) [file pbio.3003330.s012.zip › Quantification-Images/Exp-brain-1-nc82.tif]

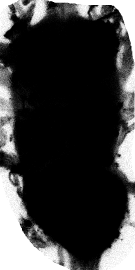

Supplement: S2 Supplemental information — (ZIP) [file pbio.3003330.s012.zip › Quantification-Images/Single-VNC-1-nc82.tif]

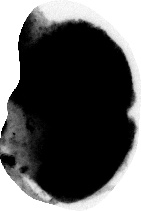

Supplement: S2 Supplemental information — (ZIP) [file pbio.3003330.s012.zip › Quantification-Images/Exp-OL-1-nc82.tif]

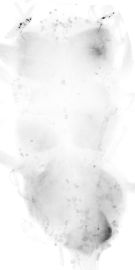

Supplement: S2 Supplemental information — (ZIP) [file pbio.3003330.s012.zip › Quantification-Images/SIngle-VNC-1-GFP.tif]

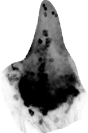

Supplement: S2 Supplemental information — (ZIP) [file pbio.3003330.s012.zip › Quantification-Images/Naive-AG-1-GFP.tif]

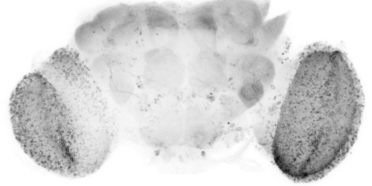

Supplement: S2 Supplemental information — (ZIP) [file pbio.3003330.s012.zip › Quantification-Images/Single-brain-1-GFP.tif]

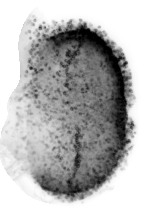

Supplement: S2 Supplemental information — (ZIP) [file pbio.3003330.s012.zip › Quantification-Images/Exp-OL-1-GFP.tif]

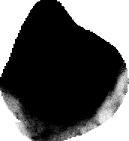

Supplement: S2 Supplemental information — (ZIP) [file pbio.3003330.s012.zip › Quantification-Images/Exp-AG-1-nc82.tif]

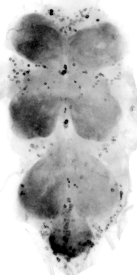

Supplement: S2 Supplemental information — (ZIP) [file pbio.3003330.s012.zip › Quantification-Images/Naive-VNC-1-GFP.tif]

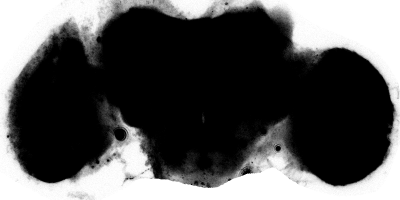

Supplement: S2 Supplemental information — (ZIP) [file pbio.3003330.s012.zip › Quantification-Images/Naive-brain-1-nc82.tif]

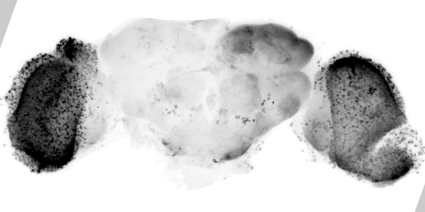

Supplement: S2 Supplemental information — (ZIP) [file pbio.3003330.s012.zip › Quantification-Images/Exp-brain-2-GFP.tif]

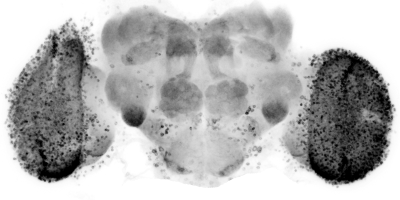

Supplement: S2 Supplemental information — (ZIP) [file pbio.3003330.s012.zip › Quantification-Images/Naive-brain-1-GFP.tif]

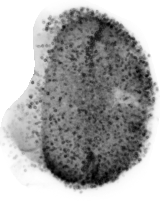

Supplement: S2 Supplemental information — (ZIP) [file pbio.3003330.s012.zip › Quantification-Images/Naive-OL-1-GFP.tif]

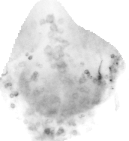

Supplement: S2 Supplemental information — (ZIP) [file pbio.3003330.s012.zip › Quantification-Images/Exp-AG-1-GFP.tif]

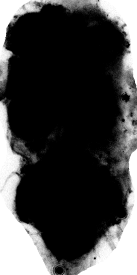

Supplement: S2 Supplemental information — (ZIP) [file pbio.3003330.s012.zip › Quantification-Images/Naive-VNC-1-nc82.tif]

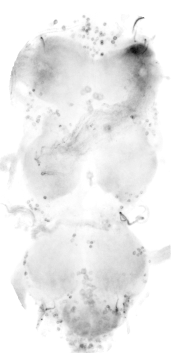

Supplement: S2 Supplemental information — (ZIP) [file pbio.3003330.s012.zip › Quantification-Images/Exp-VNC-1-GFP.tif]

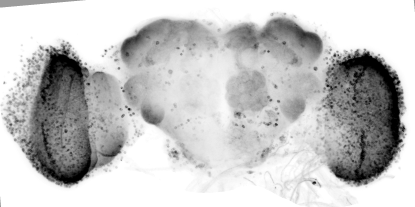

Supplement: S2 Supplemental information — (ZIP) [file pbio.3003330.s012.zip › Quantification-Images/Exp-brain-1-GFP.tif]

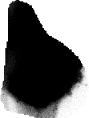

Supplement: S2 Supplemental information — (ZIP) [file pbio.3003330.s012.zip › Quantification-Images/Single-AG-1-nc82.tif]

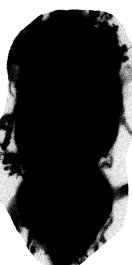

Supplement: S2 Supplemental information — (ZIP) [file pbio.3003330.s012.zip › Quantification-Images/Exp-VNC-1-nc82.tif]

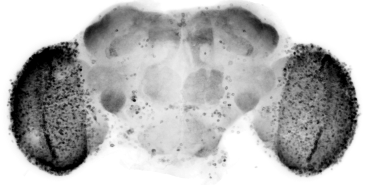

Supplement: S2 Supplemental information — (ZIP) [file pbio.3003330.s012.zip › Quantification-Images/Naive-brain-2-GFP.tif]

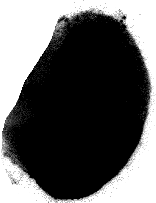

Supplement: S2 Supplemental information — (ZIP) [file pbio.3003330.s012.zip › Quantification-Images/Single-OL-1-nc82.tif]

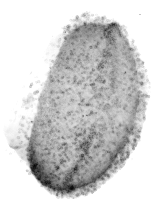

Supplement: S2 Supplemental information — (ZIP) [file pbio.3003330.s012.zip › Quantification-Images/Single-OL-1-GFP.tif]

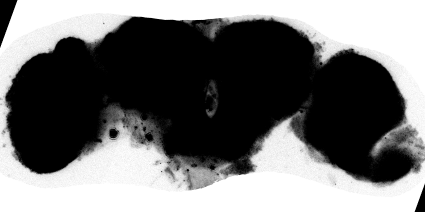

Supplement: S2 Supplemental information — (ZIP) [file pbio.3003330.s012.zip › Quantification-Images/Exp-brain-2-nc82.tif]

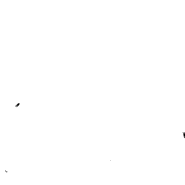

Supplement: S3 Supplemental information — (ZIP) [file pbio.3003330.s013.zip › Single-AG-1.tif]

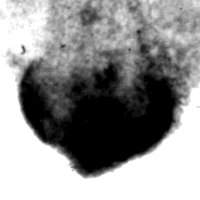

Supplement: S3 Supplemental information — (ZIP) [file pbio.3003330.s013.zip › Exp-AG-1.tif]

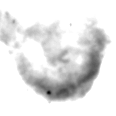

Supplement: S3 Supplemental information — (ZIP) [file pbio.3003330.s013.zip › Naive-AG-1.tif]

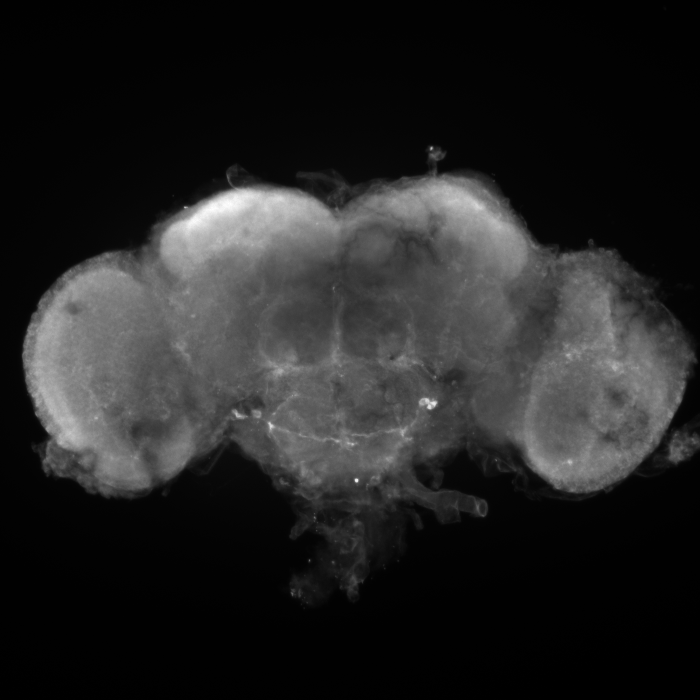

Supplement: S3 Supplemental information — (ZIP) [file pbio.3003330.s013.zip › SIFaR_ClexA_exp_brain2-fire.tif]
