# Supplementary material for: Neuropeptide-mediated synaptic plasticity regulates context-dependent mating behaviors in Drosophila
Source: PLoS Biol. 2025 Sep 4;23(9):e3003330. doi: 10.1371/journal.pbio.3003330 (PMC12410882; doi:10.1371/journal.pbio.3003330)
Supplement: S1 Table — N/A cells show that males are unable to effectively mate with females for 1 h during the mating duration assay. t-value indicate the t-statistic. df-value indicate the degrees of freedom. p-value marked in color indicate the disruption of LMD (blue) or SMD (red). Sample sizes (n) are indicated in the table. (DOCX) [file pbio.3003330.s009.docx]

**Table** **S1.** Summary of *SIFaR(24F06)-GAL4* mediated neuropeptide knockdown results

| **RNAi** **/stock** **#** | **naive** **single** **exp.** | | | | **LMD** | | **SMD** | | |
| --- | --- | --- | --- | --- | --- | --- | --- | --- | --- |
|  | *n* | *n* | *n* | *t* | *df* | *p-value* | *t* | *df* | *p-value* |
| *AKH-RNAi/* *34960* | 34 | 29 | 29 | 3.332 | 61 | 0.0015 | 1.971 | 61 | 0.0533 |
| *AKH-RNAi/* *34960* | 32 | 56 | 60 | 4.682 | 86 | <0.0001 | 2.857 | 90 | 0.0053 |
| *AstA-RNAi/* *25866* | 61 | 62 | 58 | 1.804 | 12 | 0.0737 | 0.9231 | 117 | 0.3578 |
| *Burs-RNAi/* *26719* | 64 | 57 | 44 | 2.847 | 11 | 0.0052 | 4.805 | 106 | <0.0001 |
| *Burs-RNAi/* *v13520* | 61 | 58 | 59 | 6.855 | 117 | <0.0001 | 3.730 | 118 | 0.0003 |
| *CAPA-RNAi/* *28345* | 64 | 57 | 44 | 2.847 | 119 | 0.0052 | 4.805 | 106 | <0.0001 |
| *CAPA-RNAi/* *v41124** | 67 | 60 | 0 | 1.363 | 12 | 0.1753 | N/A | N/A | N/A |
| *Crz-RNAi/* *v30670* | 56 | 44 | 45 | 1.709 | 98 | 0.0906 | 0.1890 | 99 | 0.8505 |
| *Dh44-RNAi/* *25804* | 63 | 54 | 48 | 1.665 | 115 | 0.0985 | 5.378 | 109 | <0.0001 |
| *Dh44-RNAi/* *v108473* *** | 50 | 0 | 56 | N/A | N/A | N/A | 4.403 | 104 | <0.0001 |
| *Dh44-RNAi/* *v108473* | 63 | 61 | 52 | 4.136 | 122 | <0.0001 | 3.624 | 113 | 0.0004 |
| *DSK-RNAi/* *25869* | 48 | 58 | 64 | 2.351 | 104 | 0.0206 | 1.830 | 110 | 0.0700 |
| *ETH-RNAi/* *26242* | 63 | 80 | 65 | 4.701 | 141 | <0.0001 | 3.245 | 126 | 0.0015 |
| *FMRFa-RNAi/* *58197** | 48 | 41 | 0 | 1.013 | 87 | 0.3138 | N/A | N/A | N/A |
| *LK-RNAi/* *14091** | 35 | 0 | 20 | N/A | N/A | N/A | 1.512 | 53 | 0.1366 |
| *MIP-RNAi/* *5294* | 66 | 60 | 66 | 6.318 | 124 | <0.0001 | 3.413 | 130 | 0.0009 |
| *MIP-RNAi/* *41680* | 48 | 41 | 34 | 3.651 | 87 | 0.0004 | 3.620 | 80 | 0.0005 |
| *MS-RNAi/* *24245* | 43 | 55 | 36 | 1.284 | 96 | 0.2022 | 1.707 | 77 | 0.0918 |
| *Pburs-RNAi/* *v27142* | 62 | 60 | 59 | 2.012 | 120 | 0.0465 | 2.401 | 119 | 0.0179 |
| *Proc-RNAi/* *29570* | 66 | 51 | 60 | 4.045 | 115 | <0.0001 | 2.167 | 124 | 0.0321 |
| *Proc-RNAi/* *v102488* | 63 | 61 | 60 | 3.830 | 122 | 0.0002 | 4.898 | 121 | <0.0001 |
| *TK-RNAi/* *25800* | 57 | 56 | 46 | 1.451 | 111 | 0.1496 | 3.117 | 101 | 0.0024 |
| *TK-RNAi/* *v103662* | 63 | 54 | 48 | 1.665 | 115 | 0.0985 | 5.378 | 109 | <0.0001 |

*** *Non-labeled* *(N/A)* *cells* *show* *that* *males* *are* *unable* *to* *effectively* *mate* *with* *females* *for* *1* *hour* *during* *the MD* *assay.*

*-* *Crosses* *showed* *effect* *on* *LMD/SMD* *behaviors* *are* *colored* *as* *blue* *(LMD* *defect)* *or* *red* *(SMD* *defect).*
