# Supplementary material for: Neuropeptide-mediated synaptic plasticity regulates context-dependent mating behaviors in Drosophila
Source: PLoS Biol. 2025 Sep 4;23(9):e3003330. doi: 10.1371/journal.pbio.3003330 (PMC12410882; doi:10.1371/journal.pbio.3003330)
Supplement: S2 Table — Sample sizes (n) are indicated in the table. (DOCX) [file pbio.3003330.s010.docx]

**Table** **S2.** Summary of NP-GAL4 mediated *SIFaR* knockdown results

| **RNAi** **/stock** **#** | **nai** **ve** | **single** **exp.** | | | **LMD** | | **SMD** | | |
| --- | --- | --- | --- | --- | --- | --- | --- | --- | --- |
|  | *n* | *n* | *n* | *t* | *df* | *p-value* | *t* | *df* | *p-value* |
| *AKH-GAL4/*  (Isabel 2005) | 58 | 53 | 58 | 0.825 | 109 | 0.4114 | 0.798 | 114 | 0.4266 |
| *AstA-GAL4/* *51979* | 56 | 59 | 63 | 4.217 | 113 | <0.0001 | 1.261 | 117 | 0.2100 |
| *AstC-GAL4/* *39448* | 49 | 54 | 30 | 2.743 | 101 | 0.0072 | 0.811 | 77 | 0.4201 |
| *CAPA-GAL4/* *40972* | 58 | 39 | 61 | 0.3329 | 95 | 0.7400 | 3.796 | 117 | 0.0002 |
| *Burs-GAL4/* *v13520* | 47 | 60 | 53 | 6.365 | 105 | <0.0001 | 4.133 | 98 | <0.0001 |
| *Dh31-GAL4/* *51988* | 61 | 63 | 90 | 3.262 | 122 | 0.0014 | 3.719 | 149 | 0.0003 |
| *Dh44-GAL4/* *51987* | 55 | 49 | 55 | 6.885 | 102 | <0.0001 | 2.777 | 108 | 0.0065 |
| *DSK-GAL4/* *51981* | 58 | 60 | 54 | 2.977 | 116 | 0.0035 | 4.992 | 110 | <0.0001 |
| *EH-GAL4/* *51974* | 55 | 54 | 58 | 4.034 | 107 | 0.0001 | 3.574 | 111 | 0.0005 |
| *ETH-GAL4/* *51982* | 56 | 62 | 67 | 6.882 | 116 | <0.0001 | 0.560 1 | 121 | 0.5765 |
| *FMRFa-GAL4/* *51990* | 58 | 55 | 80 | 2.194 | 111 | 0.0303 | 2.989 | 136 | 0.0033 |
| *LK-GAL4/* *51993* | 59 | 62 | 50 | 4.420 | 119 | <0.0001 | 1.060 | 107 | 0.2916 |
| *MIP-GAL4/* *51984* | 44 | 55 | 41 | 3.603 | 97 | 0.0005 | 0.928 2 | 83 | 0.3560 |
| *MS-GAL4/* *51986* | 66 | 57 | 53 | 1.119 | 121 | 0.2654 | 2.262 | 117 | 0.0256 |
| *Proc-GAL4/* *51972* | 41 | 49 | 37 | 1.746 | 88 | 0.0843 | 0.775 4 | 76 | 0.4405 |
| *sNPF-GAL4/* *46382* | 53 | 49 | 38 | 2.938 | 100 | 0.0041 | 3.799 | 89 | 0.0003 |
| *TK-GAL4/* *51974* | 48 | 56 | 49 | 3.860 | 102 | 0.0002 | 3.071 | 95 | 0.0028 |

*-* *Crosses* *showed* *effect* *on* *LMD/SMD* *behaviors* *are* *colored* *as* *sky* *blue* *(LMD* *defect)* *or*  *pink* *(SMD* *defect).*
