# Supplementary material for: Neuropeptide-mediated synaptic plasticity regulates context-dependent mating behaviors in Drosophila
Source: PLoS Biol. 2025 Sep 4;23(9):e3003330. doi: 10.1371/journal.pbio.3003330 (PMC12410882; doi:10.1371/journal.pbio.3003330)
Supplement: Attachment — Submitted filename: 147842_1_rebuttal_3225071_snxc4b.docx [file pbio.3003330.s015.docx]

**Manuscript number:** RC-2024-02546

**Corresponding author:** Woo Jae, Kim

## 1. General Statements

This is the second version of revision.

After thoroughly reviewing the comments provided by the EMBO Journal reviewers, we found their feedback to be highly constructive and valuable for enhancing our manuscript without the need for additional experiments. For example, Reviewer 1 acknowledged that our "data are intriguing and some of the experiments are quite convincing," but suggested that the manuscript contained excessive data that required simplification. This sentiment was echoed by Reviewer 2. In response, we have completely reformatted our manuscript to eliminate unnecessary imaging quantification data and CrzR-related screening data. The reviewers noted the density of our experimental data, which has led us to focus on the SIFa to Crz-CrzR circuit mechanisms related to heart function and interval timing in future projects.

Reviewer 2's comments were generally more moderate, and we successfully addressed all five of their points with detailed explanations and modifications to our manuscript. They positively remarked that "Overall, this highly interesting study advances our knowledge about the behavioral roles of SIFamide and contributes to an understanding of how motivated behavior such as mating is orchestrated by modulatory peptides." Additionally, Reviewer 3 accepted our manuscript without any further comments.

In summary, we believe we have effectively addressed all concerns raised by Reviewers 1 and 2, resulting in a clearer manuscript that is more accessible to a broader audience.

## 2. Point-by-point description of the revisions

**Reviewer #1**

***General Comments:*** *In this revision of their manuscript, Zhang et al have attempted to address most of the points raised by the reviewers, however, they have not assuaged my most important concerns. The manuscript contains a ton of information, but at times this is to the detriment of the narrative flow. I had a lot of trouble following the rationale of each experiment, and the throughline from one experiment to the next is not always obvious. The data are intriguing, and some of the experiments are quite convincing, but other experiments are either superfluous or have methodological issues. I will summarize the most acute issues below.*

**Answer:** Thank you for your thoughtful feedback and for acknowledging our efforts to address your previous comments. We appreciate your recognition of the intriguing nature of our data and the convincing aspects of our experiments. In this second revision, we have taken your concerns regarding the narrative flow and data overload to heart. We have completely reshaped our manuscript, significantly reducing unnecessary data, including the NP5270 data and overlapping quantification results that did not contribute meaningfully to the storytelling. Our goal was to streamline the presentation of our findings to enhance clarity and coherence, ensuring that each experiment clearly supports the overarching narrative. We believe these revisions will not only improve the readability of our manuscript but also allow readers to follow the rationale behind each experiment more easily. We are confident that this refined approach will make our contributions clearer and more impactful. Thank you once again for your constructive insights, which have been invaluable in guiding us toward a more focused and compelling presentation of our work.

***Comment 1.*** *The authors argue that genetic controls are unnecessary because they have been conducted in previously published papers. I am concerned with this argument, as it is good practice to repeat controls with each experiment. However, I am overall convinced by the basic phenotype indicating that panneuronal SIFaR knockdown eliminates the changes in mating duration associated with previous experience. As for the more restricted 24F06-GAL4, the phenotype is odd-the flies do actually change their mating duration, just in the opposite direction of controls. Doesn't this imply that these flies are still capable of "interval timing", and of changing their mating strategy following exposure to rivals or following sexual experience?*

**Answer:** We appreciate the reviewer’s critical comments regarding genetic control and the intriguing phenotypes we observed in specific genetic combinations. We fully agree with the reviewer and have repeated all genetic control experiments for this revision, confirming that our genetic controls consistently demonstrate intact LMD and SMD behaviors, as previously reported. These genetic control experiments have been included in Supplementary Information 1-2. We are grateful to the reviewer for the opportunity to reaffirm that LMD and SMD represent stable behavioral phenotypes suitable for genetically studying interval timing, supported by reproducible data.

We acknowledge the reviewer’s insightful comments about the exciting phenotype observed when SIFaR is knockdown which shows both singly reared and sexually experienced male show lengthened mating duration in contrast to normal LMD and SMD behaviors. Actually, we have observed such phenotype when specific neural circuits are disrupted such as when sNPF peptidergic signaling is disrupted in restricted neuronal population [4]. We are now investigating such phenotype as hypothesis as disinhibition. We explained this phenotype and about disinhibition in main text as below.

In the spatial, the targeted reduction of SIFaR expression in the GAL4^24F06^ neuronal subset resulted in a notable alteration of mating behavior. Both singly reared and sexually experienced flies exhibited an extended mating duration relative to naïve flies, contrary to the expected reduction. This observation indicates a deficit in the neural mechanism responsible for modulating mating duration, suggesting a disinhibition-like effect within the neural circuitry governing mating behavior. We have also previously observed a similar phenotype when sNPF peptidergic signaling is inhibited in specific neuronal circuits [62]. Disinhibition, characterized by the alleviation of inhibitory constraints, permits the activation of neural circuits that are ordinarily repressed. This process is instrumental in sculpting behavioral patterns and facilitating the sequential progression of behaviors. Through the orchestrated promotion of select neuronal activation and concurrent inhibition of competing neural routes, disinhibition empowers the brain with the ability to dynamically ascertain and preserve the requisite behavioral state, concurrently smoothing the transition to ensuing behavioral phases [63]. It is known that *Drosophila* neural circuits also exhibit disinhibition phenotypes in light preference and ethanol sensitization [64,65]. Further investigation is needed to uncover the underlying mechanisms of this disinhibition-like phenotype observed in LMD and SMD behaviors.

This reversed phenotype strongly suggests a disruption in interval timing, as one would expect that if interval timing were normal and intact, male flies would decrease their mating duration in response to appropriate environmental changes. For instance, research has shown that patients with Parkinson's disease exhibit heterogeneity in temporal processing, leading to disrupted interval timing phenotypes [5]. Therefore, if male flies subjected to social isolation or sexual experience do not show a reduction in mating duration compared to control conditions, it indicates a potential disruption in their interval timing mechanisms. We appreciate the reviewer’s encouragement to further explore this intriguing disinhibition-like phenotype, and we plan to investigate this aspect in our future projects.

***Comment 2.*** *I am glad the see the addition of data assessing the extent of SIFaR and CrzR RNAi knockdown; however, this has not completely addressed my concerns about interpretation of behavioral phenotypes. In both cases, the knockdown was assessed by qPCR using the very strong tub-GAL4 driver. mRNA levels are decreased but not nearly eliminated. Thus, when in line 177-178 the authors assert: "Consequently, we infer that the knockdown of SIFaR using the HMS00299 line nearly completely diminishes the levels of the SIFaR protein," the statement is not supported by the data. The qPCR results showed a knockdown at the mRNA level of ~50%. No assays were conducted to measure protein levels. The conclusions should be tempered to align with the data. Furthermore, it is not clear that knockdown is as successful with other drivers, which means that negative behavioral data must be interpreted with caution. For example, the lack of phenotype with repo-GAL4 driving SIFaR RNAi or elav-GAL4 driving CrzR RNAi could be due to a lack of efficient knockdown. This should be acknowledged.*

**Answer:** We appreciate the reviewer’s critical observation regarding the efficiency of SIFaR knockdown. We fully agree that it is essential to confirm both for ourselves and our readers that the SIFaR knockdown phenotype is robust and convincing. At the outset of this project, we tested all available SIFaR-RNAi strains following established protocols within the fly community to ensure consistency in our findings. When we employed strong drivers such as tub-GAL4 and nSyb-GAL4 for SIFaR-RNAi knockdown, we observed that the flies failed to eclose and exhibited a lethal phenotype during the larval stage, which closely resembles the homozygous lethal phenotype seen in SIFaR mutants. This suggests that, in most cases, the effects of SIFaR knockdown can effectively mimic those of SIFaR mutations. To share our methodology and reinforce our findings, we have added clarifying statements in the main text as follows:

“Employment of broad drivers, including the *tub-GAL4* and the strong neuronal driver *nSyb-GAL4*, with HMS00299 line consistently results in 100% embryonic lethality (data not shown). This phenotype mirrors the homozygous lethality observed in the *SIFaR^B322^* mutant.”

Due to the significant lethality phenotype observed, we conducted PCR analyses using a combination of *tub-GAL80^ts^* and SIFaR-RNAi. As detailed in Fig. 1E, we reared the flies at 22°C to suppress RNAi expression and then shifted the temperature to 29°C for just three days prior to performing PCR. While our PCR results indicate a 50% reduction in SIFaR levels, we believe that experiments conducted without the *tub-GAL80^ts^* system would likely demonstrate an even greater reduction in SIFaR expression. To clarify this point and provide additional context, we have included the following description in the main text:

“The silencing of SIFaR mRNA was achieved at approximately 50% using the HMS00299 knockdown line in combination with *tub-GAL80^ts^*, with RNAi induction lasting for three days (bottom diagram in Fig. 1E). Notably, the same *tub-GAL4* driver, when used without the *tub-GAL80^ts^* combination, resulted in embryonic lethality while still reducing SIFaR mRNA levels by 50% after three days of RNAi induction. This finding suggests that SIFaR knockdown using the HMS00299 line with *GAL4* drivers is likely sufficient to elicit the observed LMD and SMD behaviors. This rationale underscores the effectiveness of our experimental approach and its potential implications for understanding the role of SIFaR in mating behaviors.”

We also concur with the reviewer that the absence of a behavioral phenotype associated with CrzR-RNAi may be due to inefficient RNAi knockdown. Consequently, we have included a description of this issue in the main text as follows:

“It is important to consider that the 50% knockdown of SIFaR and CrzR may be sufficient to disrupt LMD and/or SMD behavior. However, the lack of phenotype with repo-GAL4 or elav-GAL4 could be due to a less efficient knockdown. This possibility highlights the need for cautious interpretation of negative behavioral data.”

***Comment 3.*** *Regarding the issue of outcrossing, I am confused by the authors' statement: "To reduce the variation from genetic background, all flies were backcrossed for at least 3 generations to CS strain. For the generation of outcrosses, all GAL4, UAS, and RNAi lines employed as the virgin female stock were backcrossed to the CS genetic background for a minimum of ten generations. Notably, the majority of these lines, which were utilized for LMD assays, have been maintained in a CS backcrossed state for long-term generations subsequent to the initial outcrossing process, exceeding ten backcrosses." It's not clear what this means. Perhaps the authors could definitively state how many times each line was outcrossed. The genetic background is important because of 1) the lack of all controls, and 2) the variability of the behavioral phenotype. Often, the presence or absence of LMD or SMD appears to depend on the behavior of the control flies. When these flies show low mating duration, there is typically not a reduction following sexual experience or group raising. Could these differences derive from genetic background or transgenic insertion effects?*

**Answer:** We appreciate the reviewer’s concern regarding the potential for confusion stemming from our descriptions of the genetic background. As the reviewer noted, we have published multiple papers on LMD and SMD behaviors, and we have conducted our experiments with careful attention to controlling the genetic background [1–3,6–8]. In response to the reviewer’s comments about the importance of genetic control and background, we have completed all necessary genetic control experiments and confirmed that all our flies have been backcrossed for more than ten generations to the Canton-S (CS) strain. We believe that we have adequately addressed the reviewer’s concerns regarding potential differences arising from genetic background or transgenic insertion effects. To provide readers with more detailed information about our genetic background, we have added a paragraph in the **MATERIALS AND METHODS** section as follows:

“The CS background was selected as the experimental background due to its well-characterized and consistent LMD and SMD behaviors. To ensure that genetic variation did not confound our results, all GAL4, UAS, and RNAi lines employed in our assays were rigorously backcrossed into the CS strain, often exceeding ten generations of backcrossing. This approach was undertaken to isolate the effects of our genetic manipulations from those of genetic background. We assert that the extensive backcrossing to the CS background, in concert with the internal control in LMD and SMD, provides a stable platform for the accurate interpretation of the LMD and SMD phenotypes observed in our experiments.”

***Comment 4.*** *I continue to have substantial concerns about the thresholding method used across many experiments to quantify overlap, and then to claim that this indicates that synaptic connections are being made between different neuronal populations. The degree of overlap will depend on factors including the settings during imaging (was care taken to prevent pixel saturation?). It is also not clear to me from the methods whether analysis was done on single confocal images or on projections. The images shown in the figures look like maximum projections of a confocal stack. Overlap would have to be assessed on individual confocal sections-it is possible that this is what was done for analysis but not clear from the description in the methods. Furthermore, a lot of figure space is dedicated to superfluous information. For example, in Figure 1F-J, there is a massive amount of space dedicated to assessing the agree of overlap between red stinger and CD4GFP, each driven from the same SIFaR2A driver, and further assessing what percentage of the CD4GFP signal overlaps with nc82, with the apparent goal of showing that a lot of the SIFaR signal is at active zones. This information does little to drive the narrative forward, and is quite confusing to read. Finally, the confocal images are generally too small to actually assess.*

**Answer:** We appreciate the reviewer’s concerns regarding our imaging quantification methods. We recognize the importance of providing a clear and transparent methodology for both readers and the broader scientific community. Instead of using maximum projection of confocal images, we employed a projection method that incorporates the standard deviation function available in ImageJ. Based on our experience, this approach yields more reliable quantification results, allowing for a more accurate assessment of our data. To ensure clarity and reproducibility, we have detailed our methods in the **MATERIALS AND METHODS** section as follows:

“The quantification of the overlap was performed using confocal images with projection by standard deviation function provided by ImageJ to ensure precise measurements and avoid pixel saturation artifacts.”

We appreciate the reviewer’s suggestion regarding the inclusion of image quantification data for overlapping regions, which may not be essential to the logical flow of our narrative and could lead to confusion for readers. In response, we have removed nearly all of the quantification data related to overlapping regions, retaining only those that we consider critical for the paper. Currently, only Fig. S3B-E remains, as it is important for illustrating how SIFa neuronal arborization interacts with SIFaR neurons in the central nervous system.

Additionally, we fully agree with the reviewer that the overall size of the confocal images was too small for effective assessment. To address this concern, we have enlarged all confocal images and increased the spacing in the figures. We believe these improvements will enhance the clarity of our manuscript and facilitate a better understanding of our findings.

***Comment 5.*** *In general, the figures are still very cluttered, with panels too close together, and the labels are hard to read.*

**Answer:** We thank the reviewer for their valuable feedback regarding the clarity of our figures. In response to their concern, we have enlarged the figures to enhance readability and ensure that the panels are more distinct. We believe these adjustments will significantly improve the viewer's ability to interpret the data. We appreciate the reviewer's attention to detail, which has helped us to refine the presentation of our findings.

***Comment 6.*** *There are no methodological details on how the VFB was used. The authors have not addressed my concern that they are showing only the neuronal skeleton (rather than the actual site of synapses). They are simply identifying all locations where the neuronal skeleton overlaps an entire brain region, and suggesting that these represent synapses. Many papers use the VFB to denote the actual location of synapses, which should be done in Figures 3B and S4A.*

**Answer:** We appreciate the reviewer’s constructive comments regarding the methodological details of using VFB data. We fully agree that we cannot draw definitive conclusions about SIFa projections to specific regions based solely on neuronal skeleton data, which do not indicate the actual locations of synapses. To address this concern, we have made it clear to readers that the VFB skeleton data serves only as a preliminary indication of potential SIFa projections to GA, FB, and AL.

To confirm the presence of actual synapses from SIFa neurons, we conducted a thorough analysis using FlyWire data, which validated our findings from VFB. By integrating insights from VFB with the detailed synaptic mapping provided by FlyWire, we can confidently assert the functional relevance of these connections within the context of SIFa neuronal activity. This comprehensive approach not only bolsters our conclusions but also enhances our understanding of how SIFa neurons interact within the broader neural circuitry. We believe this rationale highlights the significance of our work in elucidating the complex relationships among these neuronal populations. We have detailed our findings in the main text as follows:

“We utilized the "Virtual Fly Brain (VFB)" platform, an interactive tool designed for exploring neuronal connectivity, to gain insights into the connectivity of SIFa neurons with four other neurons, specifically GA, FB, and AL (Fig. 3B and Fig. S4B) [74]. While VFB provides valuable information, it does not offer precise locations of synapses originating from SIFa neurons. To address this limitation, we incorporated data from the FlyWire connectome, which allowed us to confirm that SIFa projections indeed form actual synapses with GA, AL, FB, and SMP (Fig. S3F and S3G) [75]. This multi-faceted approach enhances the robustness of our findings by integrating different data sources to validate neuronal connections.”

***Comment 7.*** *The changes in GRASP and CaLexA with experience are very interesting, and suggest a substantial rearrangement of synaptic connectivity associated with changes in mating duration following group rearing or female exposure. I am still concerned, however, that the nsyb and tGRASP images look so different. I wouldn't expect them to be identical, but it is puzzling that the nsyb-GRASP data show connections in a few discrete brain areas, while the tGRASP data show connections in a much larger overall brain area, but curiously not in the major regions seen with nsyb-GRASP (ie PI, FB and GA). Shouldn't the tGRASP signal appear in all the places that the nsyb-GRASP does? For CaLexA and GRASP data, the methods should indicate the timing of the dissections and staining relative to the group/sexual experience.*

**Answer:** We appreciate the reviewer’s constructive comments regarding our GRASP data, which indeed reveal an intriguing neural plasticity phenotype, as the reviewer noted. In our previous response, we suggested that the observed differences may be attributed to the distinct SIFa-GAL4 strains utilized, as described in another manuscript focused on SIFa inputs [9]. In that manuscript, we classified the four SIFa neurons into two groups: SIFa^DA^ (dorsal-lateral) and SIFa^VP^ (ventral-posterior). The *SIFa^2A^-GAL4* specifically labels only the SIFa^VP^ neurons, while the SIFa-PT driver labels all four neurons. We acknowledge that we did not clearly communicate this distinction to the reviewer or our readers, and we apologize for any confusion this may have caused. To rectify this oversight, we have added a detailed explanation of these differences in the main text as follows:

“The subtle differences in GRASP signals observed in Fig. 3A may stem from the distinct expression patterns of the SIFa^2A^-lexA and GAL4^SIFa.PT^ drivers. We would like to emphasize that the SIFa2A driver labels only a subset of SIFa neurons in other regions (Kim 2024).”

We recognize that a clear and transparent methodology is essential for generating reproducible data. In response to the reviewer’s suggestion, we have revised our **MATERIALS AND METHODS** section to include more detailed descriptions of the dissection conditions. This enhancement aims to provide readers with the necessary information to replicate our experiments effectively.

"To ascertain calcium levels and synaptic intensity from microscopic images, we dissected and imaged five-day-old flies of various social conditions and genotypes under uniform conditions. For group reared (naïve) flies, the flies were reared in group condition and dissect right after 5 days of rearing without any further action. For single reared flies, the flies were reared in single condition and dissect at the same time as group reared flies right after 5 days of rearing without any further action. For sexual experienced flies, the flies were reared in group condition after 4 days of rearing and will be given virgins to give them sexual experience for one day, those flies will also be dissected at the same time as group and single reared flies after one day."

***Comment 8.*** *The calcium imaging data are odd. In most cases, the experimental flies don't actually show an increase in calcium levels but rather a lack of a decrease that is present in the ATR- controls. Also, in the cases where they argue for an excitatory affect of SIF neuron stimulation, the baseline signal intensity appears higher in ATR- controls compared to ATR+ experimental flies (eg Fig 5L, 6O), while it is significantly higher in ATR+ flies compared to ATR- controls when the activation results in decreased calcium signals. Perhaps more details on how these experiments were conducted and whether data were normalized in some way would help to clarify this.*

**Answer:** Thank you for your valuable feedback. We appreciate your careful analysis of our calcium imaging data and have addressed your concerns below:

In our experiments, we observed that ATR+ flies maintained relatively stable calcium levels, whereas ATR- controls exhibited a gradual decrease. Under confocal imaging, GFP signals typically decrease over time, which we observed in ATR- controls. However, ATR+ flies did not exhibit this decline. To better convey this observation, we have refined the language in the manuscript. Specifically, we now describe this as a tendency to sustain the activity of Crz neurons in the OL and AG regions (Fig. 6K-M, Fig. S7G-I). This is supported by the sustained intracellular calcium activity in ATR+ flies compared to the gradual decline to baseline levels observed in ATR- controls (Fig. 6K-M).

Baseline signal intensity differences: You correctly noted that in some cases, the baseline signal intensity appears higher in ATR- controls compared to ATR+ flies. These differences are likely due to technical factors, such as variations in the distance between the imaged brain and the objective lens. Even minor positional shifts in the brain (forward or backward) can affect the observed signal intensity.

Our analyses focus on relative changes in fluorescence intensity within the same sample, which we present as line graphs to highlight trends rather than absolute values. However, we acknowledge that showing the magnitude of relative values instead of absolute values may have caused some confusion. We have revised the images to better align with our conclusions, ensuring that the adjustments do not affect the observed relative changes.

Normalization and experimental details: The calcium imaging data were normalized to ΔF/F to account for differences in baseline fluorescence intensity. However, we recognize that further clarification of the normalization process and experimental setup is essential. We have expanded the methods section to include detailed descriptions of data acquisition, normalization steps, and statistical analyses.

As the reviewer correctly noted, calcium signals in ATR^+^ flies are generally higher than those in ATR^-^ flies. However, it appears that the calcium levels exhibit a maintained response rather than a dramatic increase compared to the control ATR^-^ condition, particularly in the case shown in Fig. 6K, which illustrates SIFa-to-Crz signaling. We believe this observation may reflect the actual physiological conditions under which SIFa influences SIFaR neurons to sustain activity during activation. We have included our interpretation of these findings in the main text as follows:

“Upon optogenetic stimulation of SIFa neurons, we observed a tendency to maintain the activity of Crz neurons in OL and AG regions (Fig. 6K-M, Fig. S7G-I), evidenced by a sustained activity in intracellular Ca^2+^ levels that persisted in a high level compared to control ATR^-^ condition which shows gradual declining to baseline levels (Fig. 6K-M). In contrast to the OL and AG regions, the cells in the upper region of the SIP consistently show a decrease in Ca^2+^ levels following stimulation of the SIFa neurons (Fig. 6N-P).”

To enhance readers' understanding of our calcium imaging results, we have reformatted our GCaMP data for improved clarity and included additional details in the **MATERIALS AND METHODS** section regarding the quantification of GCaMP imaging methods. Furthermore, as the reviewer correctly noted, discrepancies in baseline activity were due to our error in presenting the baseline data. We have now corrected this oversight accordingly.

***Comment 9.*** *The models in Fig 4 J and T show data from Song et al, though I could not find a citation for this. I would omit this part of the model since these data are not discussed at all in the manuscript.*

**Answer:** We appreciate the reviewer for correctly identifying our oversight in failing to properly cite Song et al.’s paper. This error occurred partly because the preprint was not available at the time we submitted our manuscript. We now have a preprint for Song et al.’s paper, which discusses the contributions of SIFa neurons to various energy balance behaviors, and we plan to submit this paper back-to-back with our current submission to PLOS Biology. We have briefly cited Song et al.’s work in the manuscript; however, we have removed references to it from Fig. 4H and P to avoid any potential confusion for readers.

***Comment 10.*** *The graphs for the SCOPE data (eg Figure 8I-L) are still too small to make sense of.*

**Answer:** We enlarged the tSNE plot generated from the SCOPE data.

***Comment 11.*** *The rationale behind including the data in Figure 9 is not well explained. I would omit this data to help streamline and focus the manuscript.*

**Answer:** We fully understand and agree with the reviewer’s concerns, and we have removed all previous versions of Figure 9 from the manuscript to prevent any confusion regarding the storyline.

***Comment 12.*** *The single control group is still being duplicated in two different graphs but with different names in each graph. The authors updated figure caption hints at this but does not make it explicit. At the very least, these should be given the same name across all graphs, as is done, for example, in the CaLexA experiments in Figure 4B-C.*

**Answer:** We concur with the reviewer and have changed the label for all "group" conditions to "naïve" in all figures.

***Comment 13.*** *Lines 640-641: Moreover, the pacemaker function is essential for the generation of interval timing capabilities (Meck et al, 2012; Matell, 2014; Buhusi & Meck, 2005), with the heart being recognized as the primary pacemaker organ within the animal body". This is an intriguing idea, however, I attempted to look at the cited references and don't see any claim about the heart being involved in interval timing. I could not find a paper matching the citation of Matell 2014. Meck et al 2012 is an introduction to a Frontiers in Integrative Neuroscience Research Topic and does not mention the heart, nor does the Buhusi and Meck 2005 paper. Perhaps there is a more suitable reference to make the assertion that the fly's interval timer would be affected by changes in heart rate.
My suggestion would be to simplify the manuscript, focusing on the most robust findings-the behavioral effect of SIFaR knockdown, the GRASP and CaLexA data showing differences following group rearing or female exposure, and the effect of Crz knockdown in SIFaR neurons. Other details could be included but would have to be verified with more rigorous experiments.*

**Answer:** We appreciate the reviewer’s interest in our exploration of the role of heart function in interval timing. While we found that knocking down CrzR in the heart specifically disrupts LMD behavior, we agree that our manuscript needs to be streamlined for clarity. As a result, we have eliminated all CrzR-RNAi knockdown data except for the oenocyte, neuronal and glial knockdown data presented in Fig. S8C-H. This decision was made to ensure a more focused comparison with the SIFaR knockdown experiments shown in Fig. 1. We are dedicated to further investigating the role of Crz-CrzR in heart function and its influence on interval timing in a future project. This approach allows us to maintain clarity in our current manuscript while laying the groundwork for more comprehensive studies ahead.

In line with the reviewer’s suggestions, we have simplified our manuscript by eliminating unnecessary data, such as overlapping image quantification and CrzR-RNAi screening, allowing us to focus on SIFaR knockdown and GRASP, as well as CaLexA with GCaMP imaging. We are grateful to the reviewer for providing us with the opportunity to delineate the role of CrzR in heart function related to LMD as a significant future project. We believe that our manuscript has been greatly improved by the reviewer’s constructive feedback.

**Reviewer #2**

***General Comments:*** *The authors investigate mating behavior in male fruit flies, Drosophila melanogaster, and test for a role of the SIFamide receptor (SIFaR) in this type of behavior, in particular mating duration in dependence of social isolation and prior mating experience. The anatomy of SIFamide-releasing neurons in comparison with SIFamide receptor-expressing neurons is characterized in a detail-rich manner. Isolating males or exposing them to mating experience modifies the anatomical organization of SIFamidergic axon termini projecting onto SIFamide receptor-expressing neurons. This structural synaptic plasticity is accompanied by changes in calcium influx. Lastly, it is reported that corazonin-releasing neurons are modulated by SIFamide releasing neurons and impact the duration of mating behavior.

Overall, this highly interesting study advances our knowledge about the behavioral roles of SIFamide, and contributes to an understanding how motivated behavior such as mating is orchestrated by modulatory peptides. The manuscript has some points that are less convincing.*

**Answer:** We appreciate the reviewer’s positive feedback regarding our investigation into the role of the SIFamide receptor (SIFaR) in mating behavior in male *Drosophila melanogaster*. We are pleased that the detailed characterization of SIFamide-releasing neurons and their anatomical changes in response to social isolation and mating experience has been recognized as a valuable contribution to the understanding of synaptic plasticity and its impact on behavior. We are also grateful that the reviewer described our manuscript as a "highly interesting study" that advances knowledge about the behavioral roles of SIFamide and contributes to the understanding of how motivated behaviors, such as mating, are orchestrated by modulatory peptides. We sincerely thank the reviewer for these encouraging comments about our work.

We acknowledge the reviewer’s concerns about certain aspects of our manuscript that may be less convincing. We are committed to addressing these points thoroughly to strengthen our arguments and enhance the clarity of our findings. In response to the feedback, we have made several revisions throughout the manuscript, including clarifying our methodology, enhancing the presentation of our data, and providing additional context where needed. We believe these changes will improve the overall quality of the manuscript and make our conclusions more compelling. Thank you for your thoughtful review, and we look forward to your further insights.

***Comment 1.*** *It remains unclear why the authors link the differentially motivated duration of mating behavior with the psychological concept of interval timing. This distracts from the actually interesting neurobiology and is not necessary to make the study interesting. The study deals with the modulation of mating behavior by SIFamide. The abstraction that SIFamide plays a role in the neuronal calculation of time intervals for the perception of time sequenc es is not convincing in itself.*

**Answer:** We appreciate the reviewer’s thoughtful comments regarding our conclusion that links SIFamide to interval timing in mating behavior. We recognize that our data primarily indicate that SIFamide is essential for normal mating duration and influences the motivation-dependent aspects of this behavior. We also acknowledge the need for more robust evidence to establish a clearer connection between these findings and interval timing. Recent research by Crickmore et al. has provided valuable insights into how mating duration in *Drosophila* serves as an effective model for examining changes in motivation over time as behavioral goals are achieved. For example, around six minutes into mating, sperm transfer occurs, resulting in a significant shift in the male's nervous system, where he no longer prioritizes continuing the mating at the expense of his own survival. This pivotal change is mediated by four male-specific neurons that release the neuropeptide Corazonin (Crz). When these Crz neurons are inhibited, sperm transfer does not take place, and as a result, the male fails to reduce his motivation, leading to matings that can extend for hours instead of the typical duration of approximately 23 minutes [10].

Recent research conducted by Crickmore et al. has secured NIH R01 funding (Mechanisms of Interval Timing, 1R01GM134222-01) to investigate mating duration and sperm transfer timing in *Drosophila* as a genetic model for understanding interval timing. Their study emphasizes how fluctuations in motivation over time can affect mating behavior, particularly noting that significant behavioral changes occur during mating. For instance, around six minutes into the mating process, sperm transfer takes place, which corresponds with a notable decrease in the male's motivation to continue mating [10]. These findings indicate that mating duration serves not only as an endpoint for behavior but may also reflect fundamental mechanisms associated with interval timing.

We believe that by leveraging the robustness and experimental tractability of these findings, along with our own work on SIFamide's role in mating behavior, we can gain deeper insights into the molecular and circuit mechanisms underlying interval timing. We will revise our manuscript to clarify this relationship and emphasize how SIFamide may interact with other neuropeptides and neuronal circuits involved in motivation and timing.

In addition to the efforts of Crickmore's group to connect mating duration with a straightforward genetic model for interval timing, we have previously published several papers demonstrating that LMD and SMD can serve as effective genetic models for interval timing within the fly research community. For instance, we have successfully connected SMD to an interval timing model in a recently published paper [3], as detailed below:

"We hypothesize that SMD can serve as a straightforward genetic model system through which we can investigate "interval timing," the capacity of animals to distinguish between periods ranging from minutes to hours in duration.....

In summary, we report a novel sensory pathway that controls mating investment related to sexual experiences in *Drosophila*. Since both LMD and SMD behaviors are involved in controlling male investment by varying the interval of mating, these two behavioral paradigms will provide a new avenue to study how the brain computes the ‘interval timing’ that allows an animal to subjectively experience the passage of physical time [11–16]."

Lee, S. G., Sun, D., Miao, H., Wu, Z., Kang, C., Saad, B., ... & Kim, W. J. (2023). Taste and pheromonal inputs govern the regulation of time investment for mating by sexual experience in male Drosophila melanogaster. *PLoS Genetics*, *19*(5), e1010753.

We have also successfully linked LMD behavior to an interval timing model and have published several papers on this topic recently [6–8].

Sun, Y., Zhang, X., Wu, Z., Li, W., & Kim, W. J. (2024). Genetic Screening Reveals Cone Cell-Specific Factors as Common Genetic Targets Modulating Rival-Induced Prolonged Mating in male Drosophila melanogaster. *G3: Genes, Genomes, Genetics*, jkae255.

Zhang, T., Zhang, X., Sun, D., & Kim, W. J. (2024). Exploring the Asymmetric Body’s Influence on Interval Timing Behaviors of Drosophila melanogaster. *Behavior Genetics*, *54*(5), 416-425.

Huang, Y., Kwan, A., & Kim, W. J. (2024). Y chromosome genes interplay with interval timing in regulating mating duration of male Drosophila melanogaster. *Gene Reports*, *36*, 101999.

Finally, in this context, we have outlined in our INTRODUCTION section below how our LMD and SMD models are related to interval timing, aiming to persuade readers of their relevance. We hope that the reviewer and readers are convinced that mating duration and its associated motivational changes such as LMD and SMD provide a compelling model for studying the genetic basis of interval timing in *Drosophila*.

“The dimension of time is the fundamental basis for an animal's survival. Being able to estimate and control the time between events is crucial for all everyday activities [25]. The perception of time in the seconds-to-hours range, referred to as ‘interval timing’, is involved in foraging, decision making, and learning via activation of cortico-striatal circuits in mammals [26]. Interval timing requires entirely different neural mechanisms from millisecond or circadian timing [27–29]. There is abundant psychological research on time perception because it is a universal cognitive dimension of experience and behavioral plasticity. Despite decades of research, the genetic and neural substrates of temporal information processing have not been well established except for the molecular bases of circadian timing [30,31]. Thus, a simple genetic model system to study interval timing is required. Considering that the mating duration in fruit flies, which averages approximately 20 minutes, is well within the range addressed by interval timing mechanisms, this behavioral parameter provides a relevant context for examining the neural circuits that modulate the Drosophila's perception of time intervals. Such an investigation necessitates an understanding of the extensive neural and behavioral plasticity underlying interval timing [32–37].”

We would like to highlight that many researchers are currently working to bridge the gap between interval timing as a purely psychological concept and its neurobiological underpinnings, as illustrated in the following articles [15,17–20]. We appreciate the reviewer’s concerns regarding the relationship between mating duration and interval timing. However, we believe that our LMD and SMD model can effectively bridge the gap between psychological concepts and neurobiological mechanisms using a straightforward genetic model organism. By employing *Drosophila* as our model, we aim to elucidate the underlying neural circuits that govern these behaviors, thereby contributing to a deeper understanding of how interval timing is represented in both psychological and biological contexts.

Matell, M. S. Neurobiology of Interval Timing. *Adv. Exp. Med. Biol.* 209–234 (2014) doi:10.1007/978-1-4939-1782-2_12.

Matell, M. S. & Meck, W. H. Cortico-striatal circuits and interval timing: coincidence detection of oscillatory processes. *Cogn. Brain Res.* **21**, 139–170 (2004).

Merchant, H. & Lafuente, V. de. Introduction to the neurobiology of interval timing. *Adv Exp Med Biol* **829**, 1–13 (2014).

Golombek, D. A., Bussi, I. L. & Agostino, P. V. Minutes, days and years: molecular interactions among different scales of biological timing. *Philosophical Transactions Royal Soc B Biological Sci* **369**, 20120465 (2014).

Balcı, F. & Toda, K. Editorial: Psychological and neurobiological mechanisms of time perception and temporal information processing: insight from novel technical approaches. *Front. Behav. Neurosci.* **17**, 1208794 (2023).

***Comment 2.*** *For all behavioral experiments, genetic controls should always be conducted. That is, both the heterozygous Gal4-line as well as the heterozygous UAS-line should be used as controls. This is laborious, but important and common standard. The authors often report data only for offspring from genetc crosses in which UAS-lines and Gal4-lines are combined (e.g. figure S1). This is not sufficient.*

**Answer:** We are grateful for the reviewer’s constructive suggestions regarding the genetic control experiments. In response to similar concerns raised by another reviewer, we have conducted all necessary genetic control experiments and included the results in Supplementary Information 1-2. We hope that this thorough effort will demonstrate to both the reviewer and readers that the LMD and SMD behaviors represent stable and reproducible phenotypes for investigating the genetic components of interval timing.

***Comment 3.*** *There are quite a lot of citations of preprints, including preprints from the authors's own lab. It seems inappropriate to cite non-peer reviewed preprints in order to present the basic principles of the study (interval timing in flies) as recognized knowledge. In general, it is unclear whether the information presented in these multiple preprints will turn out to be credible and acceptable.*

**Answer:** We concur with the reviewer and have removed most of the preprint material, retaining only one preprint that discusses SIFa function, which has been co-submitted with this manuscript.

***Comment 4.*** *Anatomical images are often very small and not informative. For example, figure S1 O, R, S and U shows small images of fly brains and ventral nerve chords that do not convincingly describe the expression of fluorescent proteins. The choice of a threshold to quantify fluorescence seems arbitrary. It is also not clear what the quantification "83% of brain and 71% of VNC SIFaR+ neurons" actually tells us. This quantification does not rely on counting neurons (such as 83% of neurons), but only shows how fluorescence in these neurons overlaps with an immunostaining of an ubiquitous active zone protein. The same is true for figure S2 or S3: overlapping brain areas do not inform you about numbers of cells, as stated in the text.*

**Answer:** We appreciate the reviewer’s concerns regarding our imaging quantification methods. In response to similar questions raised by another reviewer, we have thoroughly reformatted our methods section and eliminated much of the overlapping data that appeared unnecessary for this paper. We recognize the importance of providing a clear and transparent methodology for both readers and the broader scientific community. Instead of using maximum projection of confocal images, we employed a projection method that incorporates the standard deviation function available in ImageJ. Based on our experience, this approach yields more reliable quantification results, allowing for a more accurate assessment of our data. To ensure clarity and reproducibility, we have detailed our methods in the **MATERIALS AND METHODS** section as follows:

“The quantification of the overlap was performed using confocal images with projection by standard deviation function provided by ImageJ to ensure precise measurements and avoid pixel saturation artifacts.”

We appreciate the reviewer’s suggestion regarding the inclusion of image quantification data for overlapping regions, which may not be essential to the logical flow of our narrative and could lead to confusion for readers. In response, we have removed nearly all of the quantification data related to overlapping regions, retaining only those that we consider critical for the paper. Currently, only Fig. S3B-E remains, as it is important for illustrating how SIFa neuronal arborization interacts with SIFaR neurons in the central nervous system.

Additionally, we fully agree with the reviewer that the overall size of the confocal images was too small for effective assessment. To address this concern, we have enlarged all confocal images and increased the spacing in the figures. We believe these improvements will enhance the clarity of our manuscript and facilitate a better understanding of our findings.

***Comment 5.*** *The authors have consistently confused the extensive overlap of neuronal processes (dendrites and presynaptic regions) across large brain areas with synaptic connections. One cannot infer functional synaptic connectivity from the overlap of these fluorescent signals.*

**Answer:** We appreciate the reviewer’s feedback and, in light of similar comments from another reviewer, we have removed most of the DenMark and syt.eGFP data, retaining only Fig. 5A. We are grateful for the constructive suggestions, which have significantly enhanced our manuscript. We believe that these revisions have clarified the narrative for readers, allowing for a more focused exploration of SIFaR's role in synaptic plasticity and neuronal orchestration.

**Reviewer #3**

***General Comments:*** *In this revised manuscript, the authors have fully and satisfactorily addressed my comments on the previous version. I recommend publication of this manuscript.*

**Answer:** We would like to extend our heartfelt thanks for the careful consideration and positive assessment of our revised manuscript. Your insightful feedback has been instrumental in shaping the final version of our work, and we are delighted to hear that our revisions have met your expectations.

Your dedication to ensuring the quality and rigor of the scientific literature is truly commendable, and we are immensely grateful for the time and effort you have devoted to reviewing our paper. Your support for publication is a significant encouragement to us and validates the hard work we have put into addressing the issues you raised.

Please accept our sincere appreciation for your professional and constructive approach throughout the review process. We look forward to the possibility of contributing to the scientific community through the dissemination of our research.

**REFERENCES**

1. Kim WJ, Jan LY, Jan YN. Contribution of visual and circadian neural circuits to memory for prolonged mating induced by rivals. Nat Neurosci. 2012;15: 876–883. doi:10.1038/nn.3104

2. Kim WJ, Jan LY, Jan YN. A PDF/NPF Neuropeptide Signaling Circuitry of Male Drosophila melanogaster Controls Rival-Induced Prolonged Mating. Neuron. 2013;80: 1190–1205. doi:10.1016/j.neuron.2013.09.034

3. Lee SG, Sun D, Miao H, Wu Z, Kang C, Saad B, et al. Taste and pheromonal inputs govern the regulation of time investment for mating by sexual experience in male Drosophila melanogaster. PLOS Genet. 2023;19: e1010753. doi:10.1371/journal.pgen.1010753

4. Zhang X, Miao H, Kang D, Sun D, Kim WJ. Male-specific sNPF peptidergic circuits control energy balance for mating duration through neuron-glia interactions. bioRxiv. 2024; 2024.10.17.618859. doi:10.1101/2024.10.17.618859

5. Merchant H, Luciana M, Hooper C, Majestic S, Tuite P. Interval timing and Parkinson’s disease: heterogeneity in temporal performance. Exp Brain Res. 2008;184: 233–248. doi:10.1007/s00221-007-1097-7

6. Sun Y, Zhang X, Wu Z, Li W, Kim WJ. Genetic Screening Reveals Cone Cell-Specific Factors as Common Genetic Targets Modulating Rival-Induced Prolonged Mating in male Drosophila melanogaster. G3: Genes, Genomes, Genet. 2024; jkae255. doi:10.1093/g3journal/jkae255

7. Zhang T, Zhang X, Sun D, Kim WJ. Exploring the Asymmetric Body’s Influence on Interval Timing Behaviors of Drosophila melanogaster. Behav Genet. 2024; 1–10. doi:10.1007/s10519-024-10193-y

8. Huang Y, Kwan A, Kim WJ. Y chromosome genes interplay with interval timing in regulating mating duration of male Drosophila melanogaster. Gene Rep. 2024; 101999. doi:10.1016/j.genrep.2024.101999

9. Kim WJ, Song Y, Zhang T, Zhang X, Ryu TH, Wong KC, et al. Peptidergic neurons with extensive branching orchestrate the internal states and energy balance of male Drosophila melanogaster. bioRxiv. 2024; 2024.06.04.597277. doi:10.1101/2024.06.04.597277

10. Thornquist SC, Langer K, Zhang SX, Rogulja D, Crickmore MA. CaMKII Measures the Passage of Time to Coordinate Behavior and Motivational State. Neuron. 2020;105: 334-345.e9. doi:10.1016/j.neuron.2019.10.018

11. Buhusi CV, Meck WH. What makes us tick? Functional and neural mechanisms of interval timing. Nat Rev Neurosci. 2005;6: 755–765. doi:10.1038/nrn1764

12. Merchant H, Harrington DL, Meck WH. Neural Basis of the Perception and Estimation of Time. Annu Rev Neurosci. 2012;36: 313–336. doi:10.1146/annurev-neuro-062012-170349

13. Allman MJ, Teki S, Griffiths TD, Meck WH. Properties of the Internal Clock: First- and Second-Order Principles of Subjective Time. Annu Rev Psychol. 2013;65: 743–771. doi:10.1146/annurev-psych-010213-115117

14. Rammsayer TH, Troche SJ. Neurobiology of Interval Timing. Adv Exp Med Biol. 2014; 33–47. doi:10.1007/978-1-4939-1782-2_3

15. Golombek DA, Bussi IL, Agostino PV. Minutes, days and years: molecular interactions among different scales of biological timing. Philosophical Transactions Royal Soc B Biological Sci. 2014;369: 20120465. doi:10.1098/rstb.2012.0465

16. Jazayeri M, Shadlen MN. A Neural Mechanism for Sensing and Reproducing a Time Interval. Curr Biol. 2015;25: 2599–2609. doi:10.1016/j.cub.2015.08.038

17. Balcı F, Toda K. Editorial: Psychological and neurobiological mechanisms of time perception and temporal information processing: insight from novel technical approaches. Front Behav Neurosci. 2023;17: 1208794. doi:10.3389/fnbeh.2023.1208794

18. Gür E, Duyan YA, Arkan S, Karson A, Balcı F. Interval timing deficits and their neurobiological correlates in aging mice. Neurobiol Aging. 2020;90: 33–42. doi:10.1016/j.neurobiolaging.2020.02.021

19. Merchant H, Lafuente V de. Introduction to the neurobiology of interval timing. Adv Exp Med Biol. 2014;829: 1–13. doi:10.1007/978-1-4939-1782-2_1

20. Matell MS. Neurobiology of Interval Timing. Adv Exp Med Biol. 2014; 209–234. doi:10.1007/978-1-4939-1782-2_12
