# Supplementary material for: Neuropeptide-mediated synaptic plasticity regulates context-dependent mating behaviors in Drosophila
Source: PLoS Biol. 2025 Sep 4;23(9):e3003330. doi: 10.1371/journal.pbio.3003330 (PMC12410882; doi:10.1371/journal.pbio.3003330)
Supplement: Attachment — Submitted filename: Point-by-point R2R_Zhang_2025_PLOS Biology.docx [file pbio.3003330.s017.docx]

**Point-by-point description of the revisions**

**Reviewer #4:**
***Major comments:***
*This manuscript by Zhang et al explores the role of SIFa signaling in regulating mating duration in Drosophila. I have looked at the comments of the reviewers on the previous version of this manuscript and assessed the revised manuscript. While I commend the authors on the different suites of methods used in this manuscript, I generally shared the concerns of the previous reviewers. Despite the revisions, it is still difficult to follow the logic behind a lot of the experiments and the conclusions from them are sometimes not supported. Below are the specific major issues with this manuscript:*

**Answer:** We sincerely thank the reviewer for their careful re-evaluation of our manuscript and for acknowledging the revisions we made in response to the previous reviewers' comments. We appreciate their recognition of the methodological approaches used in this study.

We understand and take seriously the reviewer's central concern regarding the clarity of the experimental logic and the strength of support for some conclusions. To directly address these points in this revision:

1. **Enhanced Logic & Flow:** We have undertaken a deeper restructuring of the Results and Discussion sections. We paid serious attention to explicitly stating the rationale before each key experiment or set of experiments. We ensured that the connection between the experimental question, the approach chosen, the results obtained, and the subsequent conclusions drawn is clear and unambiguous throughout the manuscript. We also strengthened the transitions between sections to improve the overall logical progression.
2. **Strengthened Support for Conclusions:** We meticulously reviewed all stated conclusions, particularly those highlighted as potentially unsupported. For each conclusion, we explicitly referenced the specific data figures/panels that support it, ensured the data presented is robust and directly relevant to the claim, toned down any language that could over-interpret the data, and more clearly acknowledged limitations or alternative interpretations where appropriate.

We believe these substantial revisions have significantly enhanced the manuscript's clarity, logical flow, and the robustness with which conclusions are supported by the data. We hope this revised version fully addresses the reviewer's valid concerns.

Below we address each comment point-by-point, with all corresponding revisions highlighted in the updated manuscript.

***Major Comment 1.***
*    *The writing is difficult to understand in a lot of places and I had to extrapolate what the authors were trying to say. I feel that the writing is not at the level expected for PLOS Biology. I provide some specific examples below.
o    "Interorgan communication is also represented by neuromodulatory signaling, which is even hormonal and conducted via the circulation."
o    "The modulating energy homeostasis between the gut and the brain is one of the most intensively studied neuromodulatory circuits via the neuronal relay of neuropeptides"
o    "The relayed event of non-synaptic and diffuse neurotransmission through neuropeptides and their receptors is also essential for the maintenance of metabolic homeostasis in Drosophila"
o    "However, the mechanism by which SIFa signaling controls various behaviors through its receptor SIFaR is not well known, because it is difficult to resolve using ordinary molecular genetics approaches and typical neural connectomics may not necessarily predict peptidergic circuits." - the authors state that it is difficult to do this and then go and use genetics tools for synaptic tracing and connectomics to find synaptic partners.*

**Answer:** We sincerely appreciate the reviewer’s feedback on improving the clarity of our writing. In response, we have carefully revised the pointed-out sentences as well as other passages that may have been unclear. We have worked to simplify phrasing, improve flow, and ensure precision in language throughout the manuscript to meet the high standards of *PLOS Biology*. We hope these edits have addressed the concerns and enhanced the overall readability of the text.

*~~"Interorgan communication is also represented by neuromodulatory signaling, which is even hormonal and conducted via the circulation."~~*

“Interorgan communication is particularly governed by neuromodulatory signaling, which operates via non-synaptic release of amines or neuropeptides. This enables both local (paracrine/volume transmission) and systemic (hormonal action via circulation) pathways to coordinate physiological responses across tissues”

*~~"The modulating energy homeostasis between the gut and the brain is one of the most intensively studied neuromodulatory circuits via the neuronal relay of neuropeptides"~~*

*~~"The relayed event of non-synaptic and diffuse neurotransmission through neuropeptides and their receptors is also essential for the maintenance of metabolic homeostasis in Drosophila"~~*

We have completely changed paragraph contains above two sentences as below:

“Neuropeptide relays represent a fundamental mechanism for energy homeostasis, particularly in gut-brain communication (Imai et al. 2009; Mayer 2011; Schoofs et al. 2016; Lin et al. 2019; Nagata and Zhou 2019; Scopelliti et al. 2019). These relays critically depend on non-synaptic transmission, where neuropeptides diffuse broadly to coordinate physiological states across tissues. While extensively documented in mammalian systems (e.g., hypothalamic integration of hunger/satiety signals via stomach, adipose, and pancreas communication (Kenny 2011; Mayer 2011)) and conserved in *Drosophila* metabolic regulation (Gao and Horvath 2008; Xu et al. 2008; Owusu-Ansah and Perrimon 2014; Yapici et al. 2014; Hentze et al. 2015; Scopelliti et al. 2019; Münch et al. 2020), the precise circuitry of central neuropeptidergic relays remains poorly characterized. This gap significantly limits our understanding of how such non-synaptic signaling modulates complex behaviors like decision-making.”

*~~"However, the mechanism by which SIFa signaling controls various behaviors through its receptor SIFaR is not well known, because it is difficult to resolve using ordinary molecular genetics approaches and typical neural connectomics may not necessarily predict peptidergic circuits." - the authors state that it is difficult to do this and then go and use genetics tools for synaptic tracing and connectomics to find synaptic partners.~~*

**Answer:** We appreciate the reviewer's insightful comment regarding our use of genetic tools for synaptic tracing and connectomics. We agree that our initial phrasing could be misinterpreted and have revised the text to clarify the specific challenges of dissecting SIFaR-mediated mechanisms. The complexity arises from two interrelated factors:

**1. Broad and Overlapping Expression of SIFaR:** Unlike synaptic neurotransmitters with discrete connectivity, SIFaR is expressed across a wide array of neuronal and non-neuronal cell types in the *Drosophila* brain. This diffuse receptor expression pattern makes it exceptionally difficult to isolate specific SIFaR-dependent circuits using conventional loss-of-function genetics or neuroanatomical tracing. Even when synaptic partners of SIFa-expressing neurons are mapped, these connections do not necessarily predict functional peptidergic transmission, as neuropeptides like SIFa primarily signal via non-synaptic volume transmission and can act on distant targets expressing the receptor .

**2. Inherent Complexity of Neuropeptide Receptor Circuit Analysis:** As highlighted in recent literature, understanding the behavioral functions of neuropeptide *receptors* lags significantly behind the characterization of neuropeptide-expressing neurons. This disparity exists because:

- **Combinatorial Complexity:** A single neuropeptide receptor (like SIFaR) integrates signals from multiple, often functionally distinct, populations of neuropeptide-expressing neurons (Taghert and Nitabach 2012; Nässel et al. 2019a; Nässel et al. 2019b).

- **Systemic Compensation:** Disrupting receptor function triggers organism-wide effects, including **homeostatic compensation** in postsynaptic cells (e.g., changes in ion channel expression or receptor sensitivity) that mask primary roles (Watteyne et al. 2024; Shao et al. 2025).

- **Pleiotropic Signaling:** Neuropeptide receptors activate slow, sustained G-protein-coupled signaling cascades that modulate neural excitability and synaptic plasticity on timescales (seconds-minutes) incompatible with traditional fast neurotransmission assays (Merighi 2017; Guillaumin and Burdakov 2021).

**Revised Manuscript Text (Excerpt):**

“The mechanisms by which SIFa signaling coordinates behavior via SIFaR remain incompletely resolved. This gap persists partly because SIFaR expression is distributed widely across diverse neural populations, complicating circuit-specific dissection. Furthermore, neuropeptide receptor circuits are inherently less tractable than those of neuropeptide-expressing neurons due to systemic compensation, volume transmission, and pleiotropic signaling modes (van den Pol 2012; Guillaumin and Burdakov 2021; Watteyne et al. 2024; Shao et al. 2025). Conventional molecular genetics and synaptic connectomics face inherent limitations in resolving neuropeptide-receptor interactions due to SIFaR's broad expression and non-synaptic signaling dynamics. To overcome these specific challenges in dissecting SIFa-SIFaR mechanisms, we employed targeted applications of these approaches—combining genetic perturbation with synaptic tracing to map functional connectivity within candidate pathways.”

We have also revised several additional sentences to enhance the clarity and readability of the manuscript, as detailed below.

*~~“Considering that the mating duration in fruit flies, which averages approximately 20 minutes, is well within the range addressed by interval timing mechanisms, this behavioral parameter provides a relevant context for examining the neural circuits that modulate the Drosophila's perception of time intervals. Such an investigation necessitates an understanding of the extensive neural and behavioral plasticity underlying interval timing.”~~*

"Fruit fly mating lasts about 20 minutes—a duration within the range of interval timing mechanisms. Thus, mating duration provides a relevant model for studying the neural circuits that govern time perception in *Drosophila*. Understanding these circuits requires insight into the neural and behavioral plasticity underlying interval timing [32-37]."

***Major Comment 2.*** *The presentation of the figures is still an issue. It is difficult to find the relevant genetic controls because they are all combined in one figure. The presentation of the confocal images (use of colors, thresholding, multiple reporters) is also questionable.*

**Answer:** We thank the reviewer for pointing this out. We would like to clarify the following in response to the concerns raised:

**1. On Genetic Controls Presentation:** We have meticulously aggregated all pertinent data and experimental circumstances related to genetic controls into the main figure of the manuscript. Upon careful evaluation of any ambiguities, we concluded that consolidating these details in the main figure would most effectively preserve clarity and avert any possible confusion for readers. By presenting the genetic control information in a cohesive and conspicuous manner, we seek to enhance the clarity and simplicity of result interpretation, ensuring that the essential experimental parameters are immediately available and easily associated with the findings mentioned in the text. We believe this technique improves the manuscript's cohesion and appreciate your understanding as we endeavor to communicate our work with clarity and precision.

**2. Use of Multiple Reporters and Color Presentation:** The use of multiple reporters (e.g., GFP, RFP, nc82) was carefully designed to highlight distinct cellular compartments. For single-channel images, grayscale was used to allow for optimal evaluation of signal strength, localization and ensuring optimal visualization for readers with color vision deficiencies. For composite images, color overlays were employed using non-overlapping pseudocolors, and the specific color assignments were labeled consistently beneath each panel. This strategy is widely used in confocal imaging to maximize signal clarity and avoid misinterpretation due to color blending.

**3.Thresholding and Signal Representation:** Thresholding was performed conservatively, with the goal of preserving weak but biologically relevant signals while avoiding oversaturation or information loss. For all quantified data, threshold settings were applied consistently across all genotypes and conditions. No nonlinear adjustments or selective signal enhancement was performed. To clarify this point, we have added a dedicated paragraph to the Methods section that outlines our image display principles, thresholding strategy, and figure preparation procedures.

We hope these improvements and clarifications address the reviewer’s concerns and help enhance the clarity and interpretability of our figures.

To enhance clarity and minimize potential reader confusion, we have included additional explanations in the **MATERIALS AND METHODS** section, as outlined below.

**“Image display and thresholding for figure preparation**

To prepare representative images for figures, grayscale channels were used for single fluorophore display to improve clarity and avoid color-based misinterpretation. For overlays involving multiple reporters (e.g., GFP, RFP, nc82), distinct non-overlapping pseudocolors were applied, with channel identities labeled below each panel. Thresholding for all displayed images was performed conservatively and consistently across genotypes and conditions, with the primary goal of preserving visible biological signals while avoiding oversaturation. For quantified data, identical threshold settings were applied across conditions. All brightness and contrast adjustments were applied uniformly to the entire image and were restricted to linear modifications for visual clarity only. No nonlinear enhancements or selective adjustments were applied. These standardized procedures ensured both accurate visualization and reproducible quantification. Raw image data are available upon request.”

Additionally, we have incorporated the following sentence into the figure legends to provide clear guidance for readers regarding our presentation.

“All images are presented using consistent thresholding across conditions, applied uniformly unless otherwise specified; further details can be found in the **MATERIALS AND METHODS** section.”

***Major Comment 3.*** *While the authors narrowed down the neurons (24F06-GAL4) that could be mediating the effects of SIFa, the precise identity of neurons expressing SIFaR important for this behavior is still lacking.*

**Answer:** We thank the reviewer for highlighting the need for greater molecular characterization of SIFaR^24F06^-expressing neurons. We have now:

1. Determined their sexual identity status: Through *dsx/fru* intersectional analysis, we show GAL4^24F06^ neurons lack canonical sex-determination markers (Supplemental information. 1A-B)
2. Defined their neuropeptidergic signatures: We identified co-expression with six neuropeptides (AstA, Capa, FMRFa, Lk, Ms, Proc) in SOG/AG neuronal populations (Supplemental information. 1C-H)
3. Established their circuit position: These neurons serve as integration hubs connecting SIFa inputs to multiple peptidergic pathways

This molecular profiling (summarized in new Supplemental information. 1) significantly advances our understanding beyond initial anatomical identification by:

- Revealing their non-canonical sexual identity
- Demonstrating multiplexed neuropeptide co-expression
- Positioning them within a functional network for behavioral timing

While single-cell resolution would be ideal, this comprehensive molecular characterization provides critical insights into the identity and functional organization of these SIFaR-expressing neurons.

We have added additional data and descriptions as below:

“To further characterize the molecular identity of *GAL4^24F06^* neuronal population, we first examined potential sexual dimorphism using intersectional approaches with dsxFLP and fruFLP based fluorescent reporters. Surprisingly, no *GAL4^24F06^* neurons expressed dsx or fru (Supplemental information. 1A-B), indicating these neurons lack canonical sexual identity markers. We next mapped neuropeptide co-expression patterns using *lexA^24F06^* and *GAL4* lines for peptidergic pathways known to modulate LMD/SMD behaviors (Wong 2019). Strikingly, in the SOG and AG, *lexA^24F06^* neurons showed extensive co-localization with six neuropeptides: AstA, Capa, FMRFa, Lk, Ms, and Proc (Supplemental information. 1C-H). These findings suggest that: (1) the SOG and AG serve as critical integration hubs where *GAL4^24F06^* neurons receive SIFa inputs from the PI region, and (2) these neurons may coordinate interval timing decisions across social-sexual contexts through regulation of these neuropeptidergic pathways.” (line 242-254)

***Major Comment 4.*** *The functional connectivity data using GCAMP are not convincing due to the decay in signal in some controls but not others. Calcium signals should not drop this drastically within the 60 seconds presented in the figures.*

**Answer:** We sincerely appreciate the reviewer’s careful attention to the GCaMP signal dynamics in our functional connectivity experiments. Upon re-examining our original dataset and repeating the control measurements, we identified and corrected technical inconsistencies in the signal acquisition that affected some control recordings. The new analyses (now presented in Fig. 5J and Fig. 7B) demonstrate stable calcium signals throughout the 60-second observation window across all control groups. These revised data confirm the robustness of our experimental conditions and validate the differential responses we observed in experimental groups.

***Specific comments***
***Comment 1.*** *Line 100: SIFa is expressed in more than 4 neurons and includes neurons that innervate the central complex. It does have strongest expression in the 4 neurons.*

**Answer:** We thank the reviewer for highlighting this important point. Researchers have long investigated the anatomical characteristics of SIFa neurons across insect species, including *Drosophila*. The first report by Verleyen et al. (2004) demonstrated that SIFamide is a highly conserved neuropeptide, with clear evidence of four cell bodies in the pars intercerebralis (PI) of *N. bullata*, *A. gambiae*, *A. alni*, and *D. melanogaster*. While Verleyen et al. noted that *Drosophila* "most likely" has four SIFa neurons, subsequent studies—including Terhzaz et al. (2007), Park et al. (2014), Sellami et al. (2015), and Martelli et al. (2017)—definitively confirmed that *D. melanogaster* possesses **four large PI neurons expressing SIFa**, which project broadly across the CNS, including the central complex (Verleyen et al. 2004; Terhzaz et al. 2007a; Park et al. 2014; Sellami and Veenstra 2015a; Martelli et al. 2017).

**1. Four Neurons, Conserved Across Species:**

- The four PI neurons are **the exclusive source** of SIFa in *Drosophila*, as supported by genetic, anatomical, and transcriptomic studies (Park et al. 2014; Martelli et al. 2017).

- Immunoreactive signals in other regions (e.g., central complex) reflect **axonal transport** from these four somata, not additional expression sites (Terhzaz et al. 2007a; Sellami and Veenstra 2015a).

**2. Clarifying Potential Misinterpretations:**

- While some anti-SIFa sera may detect faint immunoreactivity in projections, this likely represents peptide transport rather than active synthesis. No studies report additional SIFa-producing cell bodies beyond the four PI neurons.

- Functional experiments (e.g., optogenetics in Park et al., 2014) exclusively target these four neurons to modulate central complex circuits, further confirming their role as the sole source (Park et al. 2014; Huang et al. 2021).

To improve clarity, we have added new sentences and citation in main text as below:

“SIFa is synthesized in four PI neurons and transported to the broad regions of CNS including central complex via axonal projections [38–42].”

***Comment 2.*** *Figure S1A: How can prior exposure to competing males be regarded as naive males? Shouldn't it be referred to as grouped to distinguish from single?*

**Answer:** We agree with the reviewer that the term *"naive"* could be misleading in this context, as prior exposure to competing males may influence behavior. In the revised manuscript and figures, we have replaced *"naive"* with *"group"* throughout to clearly distinguish these males from those raised in isolation (*"single"*). We appreciate this clarification and have updated the text and figures accordingly.

***Comment 3.*** *Line 130-133: "The SIFaR, the receptor for SIFa, has been linked to circadian rhythm, feeding, courtship, sleep, and memory extinction; however, its functional properties remain poorly understood". If those are the functions of SIFaR then how are its "functional properties" poorly understood?*

**Answer:** As addressed in our response to the reviewer’s major **Comment 1**, we have revised the relevant sentences to better clarify the challenges associated with dissecting SIFaR-expressing circuits.

“The mechanisms by which SIFa signaling coordinates behavior via SIFaR remain incompletely resolved. This gap persists partly because SIFaR expression is distributed widely across diverse neural populations, complicating circuit-specific dissection. Furthermore, neuropeptide receptor circuits are inherently less tractable than those of neuropeptide-expressing neurons due to systemic compensation, volume transmission, and pleiotropic signaling modes [10,49–51]. Conventional molecular genetics and synaptic connectomics face inherent limitations in resolving neuropeptide-receptor interactions due to SIFaR's broad expression and non-synaptic signaling dynamics. To overcome these specific challenges in dissecting SIFa-SIFaR mechanisms, we employed targeted applications of these approaches—combining genetic perturbation with synaptic tracing to map functional connectivity within candidate pathways.”

***Comment 4.*** *Line 141-142: It is extremely difficult to find the relevant genetic controls for the experiments presented in Figure 1A-D. Supplemental information. 1 is just one figure containing genetic controls from different experiments. I dont understand why the authors have chosen this format for presenting the data.*

**Answer:** We appreciate the reviewer’s attention to methodological rigor. In accordance with their suggestion, we have now included all relevant genetic controls in the main figures to ensure clarity and accessibility for readers. This adjustment provides a more comprehensive presentation of the data while maintaining the logical flow of the results.

***Comment 5.*** *Figure 1F-G: It is extremely difficult to make any sense of the SIFaR expression pattern based on current presentation. Compared to the staining pattern for the same driver depicted in the chemoconnectome manuscript, none of the images add anything new.*

**Answer:** We appreciate the reviewer's constructive feedback on the SIFaR expression data. In response:

**1. Removal of redundant data**: We have deleted the brain imaging panels (original Fig. 1F) since these largely recapitulate published patterns.

**2. Focus on novel VNC findings**: The retained VNC images (revised Fig. 1L) reveal distinct SIFaR expression.

Since the original chemoconnectome study did not characterize SIFaR neuron projections in the ventral nerve cord (VNC), we have now clarified this novel finding in our main text as follows:

“We used the newly developed chemoconnectome (CCT) knock-in SIFaR-GAL4 (*SIFaR^2A-AC.GAL4^, SIFaR^2A^* hereafter) strain [61] to examine SIFaR expression in the nervous system, while the expression pattern of SIFaR in the brain has been previously characterized [61], our findings reveal abundant SIFaR expression throughout the VNC highly overlapping with neuropil marker nc82 (Fig. 1L).”

***Comment 5.*** *Lines 173-178: Previous reviewers have all critiqued the interpretation of nc82 and GFP staining. I agree with them. The authors over interpreting their findings. The only conclusion that can be made from their staining is that SIFaR is broadly expressed in the CNS.*

**Answer:** We thank the reviewer for emphasizing the need for precise interpretation of our nc82/GFP colocalization data. We fully agree that these results alone demonstrate broad SIFaR expression in the CNS and should not be overextended to imply functional roles without further validation.

To address this concern, we have revised the text (Lines 183-194) as follows:

“Neuropil is a synaptic dense region in the nervous system composed of mostly unmyelinated axons, dendrites and glial cell processes [62]. The monoclonal antibody nc82, which identifies *Drosophila* Bruchpilot (Brp), a constituent of the CAST1/ERC family and a component of the active zone, has been employed to examine the localization of certain neuronal populations. The significant overlap between the membrane marker of these neurons and the neuropil marker nc82 indicates a possible presence at the active zone [63]; nonetheless, this should not be construed as direct evidence of functionality. The studies demonstrate that SIFaR is extensively expressed in the CNS. Although the expression pattern is indicative, it is crucial to acknowledge that additional tests are necessary to confirm a direct correlation between SIFaR expression in these neurons and the initiation of interval timing behaviors.”

***Comment 6.*** *Line 184: replace "promoter" with "regulatory" since two drivers target an intronic region which is not normally a "promoter".*
 **Answer:** We thank the reviewer for this precise technical suggestion. We have replaced all instances of "promoter" with "regulatory" when referring to the targeted intronic regions in our driver lines, as this more accurately describes their non-canonical genomic location. We agree this terminology better reflects the molecular nature of these genetic tools.

***Comment 7.*** *Line 187-189: "In the spatial, the targeted reduction of SIFaR expression in the GAL4^24F06^ neuronal subset resulted in a notable alteration of mating behavior." - I dont understand what in the spatial means.*
 **Answer:** We appreciate the reviewer catching this unclear phrasing. We've removed the confusing term 'in the spatial' and revised the text to clearly state that we observed behavioral effects when manipulating SIFaR specifically in the *GAL4^24F06^* neuronal population:

"Targeted reduction of SIFaR expression specifically in the *GAL4^24F06^* neuronal subset resulted in altered mating behavior."

This change better communicates our experimental approach while maintaining scientific precision.

***Comment 8.*** *Figure 2 results: The whole discussion on disinhibition is long, speculative and out of place in the results section.*

**Answer:** We agree with the reviewer that the discussion of disinhibition was too extensive and speculative for the Results section. We have now:

1. Reduced the main text description of disinhibition mechanisms in the Results section, keeping only the essential experimental findings.
2. Moved the more detailed discussion of potential disinhibition effects to the Discussion section, where we properly frame it as a hypothesis requiring future validation.

This change better aligns with standard manuscript organization and prevents overinterpretation of results in the data presentation section.

In the main text:

“The prolonged mating duration observed after *SIFaR* knockdown suggests a disinhibition-like effect within the mating circuitry. This phenotype is similarly evident in *Drosophila* light preference and ethanol sensitization behaviors, indicating a broader regulatory role of *SIFaR* in inhibitory control.”

In the Discussion section:

“Knockdown of SIFaR in *GAL4^24F06^*-expressing neurons induces a disinhibition-like phenomenon. Disinhibition, characterized by the alleviation of inhibitory constraints, permits the activation of neural circuits that are ordinarily repressed. This process is instrumental in sculpting behavioral patterns and facilitating the sequential progression of behaviors. Through the orchestrated promotion of select neuronal activation and concurrent inhibition of competing neural routes, disinhibition empowers the brain with the ability to dynamically ascertain and preserve the requisite behavioral state, concurrently smoothing the transition to ensuing behavioral phases [83]. It is known that Drosophila neural circuits also exhibit disinhibition phenotypes in light preference and ethanol sensitization [66,67]. Further investigation is needed to uncover the underlying mechanisms of this disinhibition-like phenotype observed in LMD and SMD behaviors.”

***Comment 9.*** *Figure 2F: What is the point of having greyscale and colored images for the same staining? The reporter in the figure is red stinger but the images are of GFP staining from FlyLight. I am not sure if FlyLight images can be used in publication without approval. I also dont understand the thresholding in Figure 2G. Why is not sufficient to just show the staining pattern in males and females without commenting on differences in numbers which were not properly quantified?*

**Answer:** We sincerely appreciate the reviewer's valuable feedback. To enhance accessibility, we have included both grayscale and color versions of same staining images, to allow for optimal evaluation of signal strength, localization and ensuring optimal visualization for readers with color vision deficiencies.

We have also carefully replaced all FlyLight reference images with our own experimental data to maintain consistency throughout the manuscript.

We acknowledge that 'Redstinger' was incorrectly stated in our previous response - this has been corrected to accurately reflect our experimental system.

Regarding Figure 2G, we have removed the thresholding analysis to avoid potential misinterpretation, as our goal was to qualitatively illustrate expression patterns rather than make quantitative claims about sexual dimorphism. These modifications have strengthened both the clarity and accuracy of our presentation.

***Comment 10.*** *Figure S2G: It would have been much informative if the authors had used a FLP based fluorescence reporter to show only the common neurons between the two lines. At the moment it is not possible to see any detailed morphology about the neurons that are common in both drivers.*
 **Answer:** We thank the reviewer for this insightful suggestion. In response, we have now performed the recommended FLP-out experiment to specifically label the overlapping neurons between the two driver lines (GAL4^24F06^ and lexA^SIFaR.2A^). These new data are presented in Figure S2H, which clearly reveals the detailed morphology of the shared neuronal population. This addition significantly strengthens our analysis by precisely identifying the common neural circuitry underlying our observed phenotypes.

“To better characterize the co-expression of *GAL4^24F06^* and *SIFaR^2A^*, we employed an FLP-dependent fluorescence reporter system. These results (Fig. S2H) confirm and extend our previous findings, demonstrating consistent overlap between these genetic markers in OL, SOG and AG.”

***Comment 11.*** *Line 231.243: The switch from LMD/SMD to feeding does not make sense and it is out of place. It is just results that don’t fit in with the rest of the story.*

**Answer:** We thank the reviewer for their valuable critique regarding the feeding behavior data. After careful consideration, we fully agree that these observations disrupted the logical flow of our study focused on interval timing behavior. Consequently, we have removed all feeding-related results from the manuscript. This revision has significantly improved the paper's coherence by maintaining a clear focus on our central findings regarding the affection of neuropeptide relay of SIFa-SIFaR on mating behavior in *Drosophila*. The streamlined presentation now better highlights the conceptual continuity of our work.

***Comment 12.*** *Line 247-260: As mentioned by previous reviewers, I also don’t understand the reasoning for doing this kind of colocalization analysis. Not all 24F06-GAL4 neurons express SIFaR so I am not sure what this analysis adds. For the same reasons, I think that conclusions based on Figure 3 are premature - the authors have not shown that all the 24F06-GAL4 neurons are SIFaR positive. Therefore, synapses could be formed by neurons that don’t express SIFaR.  The images in Figure S3D are too small.*

**Answer:** We appreciate the reviewer's valid concern about heterogeneity in the *24F06-GAL4* population. To clarify our rationale and address these points:

**1. Purpose of colocalization analysis**: This was not intended to imply all *24F06-GAL4* neurons express SIFaR, but rather to **identify *potential* neuroanatomical hubs** where SIFa/SIFaR signaling may occur. As noted in our revised text (Lines 272-275):

"While *GAL4^24F06^* labels diverse neurons, the partial overlap with SIFaR highlights specific subregions (e.g., SOG and AG) where SIFa-SIFaR interactions are anatomically plausible, guiding future cell-type-specific investigations."

We appreciate the reviewer's question about SIFaR function in 24F06-GAL4 neurons. Our key evidence comes from rescue experiments showing that expressing SIFaR specifically in just these neurons completely rescues both the lethal phenotype and behavioral deficits (LMD and SMD) of SIFaR mutants. This demonstrates that SIFaR expression in 24F06-GAL4 neurons is both necessary and sufficient for these functions. While we agree that not all 24F06-GAL4 neurons show SIFaR expression (as noted in our colocalization analysis), the complete rescue by this driver strongly indicates that the SIFaR-positive subset within this population mediates these essential functions. We've clarified this point in the revised discussion to better connect the anatomical and functional data.

**2. Revised conclusions for Figure 3**: We now explicitly acknowledge heterogeneity and focus on **circuit-level implications** rather than universal expression (line 277-283):

"The observed synaptic alterations in *GAL4^24F06^* neurons may arise from the integrated activity of both SIFaR-expressing and non-expressing subpopulations. Nevertheless, given the functional significance of *GAL4^24F06^* neurons in rescuing lethality (Table. 1) and behavioral deficits (Fig. 2F-G) in genetic complementation assays, our data support a model wherein SIFa-SIFaR signaling modulates interval timing behaviors through dual mechanisms: direct synaptic modulation and indirect network-level integration.”

In addition, our experimental results demonstrate that SIFaR-expressing neurons labeled by GAL4^24F06^ in the SOG and AG exhibit differential sensitivity to various social and sexual conditions. Through multiple complementary approaches - including SIFa-SIFaR-tGRASP interaction mapping, CalexA neural activity monitoring, and syt.eGFP synaptic labeling - we consistently observed condition-dependent modulation of these neural populations. These findings strongly suggest that SIFaR signaling in these specific neuropil regions plays a critical role in regulating interval timing behaviors.

**3. Addressing Figure S3D**: We have enlarged all panels in Figure S3D by 200% and added insets highlighting key regions of interest for clarity.

***Comment 13.*** *Figure S3F and G: The authors claim that they used the FlyWire connectome to do the analyses but the IDs provided are for SIFa neurons in hemibrain which is a completely different dataset. Please clarify what dataset was actually used for the analyses.*

**Answer:** We thank the reviewer for catching this mistake. The mention of "FlyWire" in the main text was an error. Both the figure and the methods correctly refer to the FlyEM hemibrain connectome (v1.2.1) on VFB. The text has been corrected accordingly.

“To address this limitation, we incorporated data from the FlyEM hemibrain connectome (v1.2.1)”

***Comment 14.*** *Figure 4A-H. What is the expression pattern of the GAL4 used in these experiments? My issue with GRASP is that if SIFaR expression is altered under different contexts, it will directly affect the amount of reporter signal since this is a LexA knock-in line. This could affect the GRASP signal even if the number of synapses remains constant.*

**Answer:** We appreciate the reviewer's insightful comment regarding the GAL4 expression pattern and GRASP interpretation. To clarify:

1. The GAL4 driver used in these experiments (GAL4^SIFa.PT^) shows expression in elsewhere (Terhzaz et al. 2007b; Sellami and Veenstra 2015b).

2. The lexA driver used in these experiments are SIFaR.2A-lexA, the expression pattern was show in Fig. S2G.

3. Regarding GRASP signal variability: We acknowledge that changes in SIFaR-LexA expression levels across contexts could influence GRASP signals. However, this precisely reflects our key finding - that different social contexts dynamically regulate:

a) SIFaR neuronal activity

b) SIFa-SIFaR signaling pathway engagement

The GRASP signal changes thus represent biologically meaningful modulation of this pathway

***Comment 15.*** *Figure 4P: The schematic needs to be altered as not all 24F06-GAL4 neurons express SIFaR. Hence, some of these cells could also be activated by other neuropeptides/neurotransmitters besides SIFa.*

**Answer:** We thank the reviewer for this important insight. We have implemented two key revisions to address the concern that not all 24F06-GAL4 neurons express SIFaR:

1. **Schematic Revision (Figure 4P):** The diagram now explicitly distinguishes **SIFaR-expressing subpopulations** (solid color) from other 24F06-GAL4 neurons (outline only).
2. **Discussion Amendment (Lines 556-565):**

“Although our study reveals a key role for the SIFa-SIFaR pathway in regulating interval timing, the *GAL4^24F06^*-labeled neuronal population likely integrates additional neuromodulatory and sensory inputs. The observed behavioral effects may thus reflect a convergence of signals beyond SIFa alone. Future studies combining single-cell transcriptomics to resolve molecular heterogeneity with subpopulation-specific functional manipulations (e.g., intersectional genetics or input mapping) could delineate how distinct circuit elements process these diverse inputs to collectively govern temporal control. Such approaches would clarify whether functional subdivisions exist within this population and how they contribute to behavioral flexibility across contexts.”

***Comment 16.*** *Figure 5H-L: There are several neurons in the AG that are labelled by the 24F06 GAL4. In fact, most of the AG seems stained with GFP in Figure 2F. So which neurons was GCAMP signal measured in? The representative figure in Figure 5L seems to show very few pixels and the number of neurons cannot be estimated from that image. I also share the concern with the previous reviewer that the calcium signal in Figure 5J should not decay so fast (within a minute) in the control. I am not sure if this is because the cell was not functioning normally.*
 **Answer:** We sincerely appreciate the reviewer's meticulous critique. We have implemented the following improvements:

1. **Whole AG imaging with cellular resolution** (revised Fig. 5L): The updated figure now displays the entire AG region *GAL4^24F06^* neurons. Arrows indicate analyzed cells.
2. We reperformed the control experiment and analyzed, the new analysis demonstrates stable baseline signals throughout the recording period.

***Comment 17.*** *Lines 405-406: It is known that no Crz peptide is produced in the OL. This is due to post-transcriptional regulation of Crz mRNA.* (<https://pubmed.ncbi.nlm.nih.gov/18087727/)>.

**Answer:** We appreciate the reviewer’s comment regarding Crz peptide production in the OL. We agree that Crz peptide may be absent in OL due to post-transcriptional regulation (Lee et al. 2008). Following reviewer’s suggestion, we have modified main text as below:

“Given the established absence of Crz peptide in the OL [81], SIFaR^24F06^-expressing neurons within this region may represent a critical neural substrate for LMD behavior that operates independently of Crz signaling.”

***Comment 18.*** *Figure 6K-L: sample size of 3 is not sufficient. The decay in calcium signal in control is an issue again especially since the control in Figure 6N looks completely stable over the imaging period.*

**Answer:** We sincerely thank the reviewer for identifying these critical issues. We have addressed them as follows:

1. Increased sample size: The calcium imaging experiments (originally n=3) were repeated to reach n=6 independent biological replicates per condition. The updated Fig. 7B-D demonstrates consistent effects across all trials.

2. Resolved control variability: The revised data show stable control baselines, aligning with Fig. 7E.

***Comment 19.*** *Lines 443-445: This conclusion is not fully supported by the data for the reasons highlighted above.*

**Answer:** We thank the reviewer for this constructive suggestion. We agree our original conclusion overstated the comprehensiveness of the SIFa-SIFaR/Crz-CrzR mechanism and have revised it to better reflect the anatomical constraints (particularly Crz's absence in the optic lobe) while emphasizing the abdominal ganglion's role. We changed our main text as below (Line 470-473):

“Moreover, these results support a model where long-range neuropeptide signaling - specifically SIFa-SIFaR in the brain coupled with Crz-CrzR in the abdominal ganglion - contributes significantly to the neural mechanisms governing interval timing (Fig. 7H).”

**REFERENCES**

Gao Q, Horvath TL. 2008. Neuronal control of energy homeostasis. Febs Lett. 582(1):132–141. doi:10.1016/j.febslet.2007.11.063.

Guillaumin MCC, Burdakov D. 2021. Neuropeptides as Primary Mediators of Brain Circuit Connectivity. Front Neurosci. 15:644313. doi:10.3389/fnins.2021.644313.

Hentze JL, Carlsson MA, Kondo S, Nässel DR, Rewitz KF. 2015. The Neuropeptide Allatostatin A Regulates Metabolism and Feeding Decisions in Drosophila. Sci Rep-uk. 5(1):11680. doi:10.1038/srep11680.

Huang H, Possidente DR, Vecsey CG. 2021. Optogenetic activation of SIFamide (SIFa) neurons induces a complex sleep-promoting effect in the fruit fly Drosophila melanogaster. Physiol Behav. 239:113507. doi:10.1016/j.physbeh.2021.113507.

Imai J, Oka Y, Katagiri H. 2009. Identification of a novel mechanism regulating β-cell mass: Neuronal relay from the liver to pancreatic β-cells. Islets. 1(1):75–77. doi:10.4161/isl.1.1.8615.

Kenny PJ. 2011. Common cellular and molecular mechanisms in obesity and drug addiction. Nat Rev Neurosci. 12(11):638–651. doi:10.1038/nrn3105.

Lee G, Kim K-M, Kikuno K, Wang Z, Choi Y-J, Park JH. 2008. Developmental regulation and functions of the expression of the neuropeptide corazonin in Drosophila melanogaster. Cell Tissue Res. 331(3):659–673. doi:10.1007/s00441-007-0549-5.

Lin S, Senapati B, Tsao C-H. 2019. Neural basis of hunger-driven behaviour in Drosophila. Open Biol. 9(3):180259. doi:10.1098/rsob.180259.

Martelli C, Pech U, Kobbenbring S, Pauls D, Bahl B, Sommer MV, Pooryasin A, Barth J, Arias CWP, Vassiliou C, et al. 2017. SIFamide Translates Hunger Signals into Appetitive and Feeding Behavior in Drosophila. Cell Reports. 20(2):464–478. doi:10.1016/j.celrep.2017.06.043.

Mayer EA. 2011. Gut feelings: the emerging biology of gut–brain communication. Nat Rev Neurosci. 12(8):453–466. doi:10.1038/nrn3071.

Merighi A. 2017. Reference Module in Neuroscience and Biobehavioral Psychology. (Neuropharmacology 39 2000). doi:10.1016/b978-0-12-809324-5.02310-5.

Münch D, Ezra-Nevo G, Francisco AP, Tastekin I, Ribeiro C. 2020. Nutrient homeostasis — translating internal states to behavior. Curr Opin Neurobiol. 60:67–75. doi:10.1016/j.conb.2019.10.004.

Nagata S, Zhou YJ. 2019. Feeding-modulating neuropeptides and peptide hormones in insects. Adv Insect Physiol.:137–172. doi:10.1016/bs.aiip.2019.10.002.

Nässel DR, Pauls D, Huetteroth W. 2019a. Neuropeptides in modulation of Drosophila behavior: how to get a grip on their pleiotropic actions. Curr Opin Insect Sci. 36(eLife 7 2018):1–8. doi:10.1016/j.cois.2019.03.002.

Nässel DR, Pauls D, Huetteroth W. 2019b. Neuropeptides in modulation of Drosophila behavior: how to get a grip on their pleiotropic actions. Peerj Prepr. 7:e27531v1. doi:10.7287/peerj.preprints.27531v1.

Owusu-Ansah E, Perrimon N. 2014. Modeling metabolic homeostasis and nutrient sensing in Drosophila: implications for aging and metabolic diseases. Dis Model Mech. 7(3):343–350. doi:10.1242/dmm.012989.

Park S, Sonn JY, Oh Y, Lim C, Choe J. 2014. SIFamide and SIFamide Receptor Define a Novel Neuropeptide Signaling to Promote Sleep in Drosophila. Mol Cells. 37(4):295–301. doi:10.14348/molcells.2014.2371.

Schoofs L, Loof AD, Hiel MBV. 2016. Neuropeptides as Regulators of Behavior in Insects. Annu Rev Entomol. 62(1):35–52. doi:10.1146/annurev-ento-031616-035500.

Scopelliti A, Bauer C, Yu Y, Zhang T, Kruspig B, Murphy DJ, Vidal M, Maddocks ODK, Cordero JB. 2019. A Neuronal Relay Mediates a Nutrient Responsive Gut/Fat Body Axis Regulating Energy Homeostasis in Adult Drosophila. Cell Metab. 29(2):269-284.e10. doi:10.1016/j.cmet.2018.09.021.

Sellami A, Veenstra JA. 2015a. SIFamide acts on fruitless neurons to modulate sexual behavior in Drosophila melanogaster. Peptides. 74:50–56. doi:10.1016/j.peptides.2015.10.003.

Sellami A, Veenstra JA. 2015b. SIFamide acts on fruitless neurons to modulate sexual behavior in Drosophila melanogaster. Peptides. 74:50–56. doi:10.1016/j.peptides.2015.10.003.

Shao J, Liewald JF, Costa WS, Ruse C, Gruber J, Djamshedzad MS, Gebhardt W, Gottschalk A. 2025. Loss of neuropeptidergic regulation of cholinergic transmission induces homeostatic compensation in muscle cells to preserve synaptic strength. PLOS Biol. 23(5):e3003171. doi:10.1371/journal.pbio.3003171.

Taghert PH, Nitabach MN. 2012. Peptide Neuromodulation in Invertebrate Model Systems. Neuron. 76(1):82–97. doi:10.1016/j.neuron.2012.08.035.

Terhzaz S, Rosay P, Goodwin SF, Veenstra JA. 2007a. The neuropeptide SIFamide modulates sexual behavior in Drosophila. Biochem Bioph Res Co. 352(2):305–310. doi:10.1016/j.bbrc.2006.11.030.

Terhzaz S, Rosay P, Goodwin SF, Veenstra JA. 2007b. The neuropeptide SIFamide modulates sexual behavior in Drosophila. Biochem Biophys Res Commun. 352(2):305–310. doi:10.1016/j.bbrc.2006.11.030.

van den Pol AN. 2012. Neuropeptide Transmission in Brain Circuits. Neuron. 76(1):98–115. doi:10.1016/j.neuron.2012.09.014.

Verleyen P, Huybrechts J, Baggerman G, Lommel AV, Loof AD, Schoofs L. 2004. SIFamide is a highly conserved neuropeptide: a comparative study in different insect species. Biochem Bioph Res Co. 320(2):334–341. doi:10.1016/j.bbrc.2004.05.173.

Watteyne J, Chudinova A, Ripoll-Sánchez L, Schafer WR, Beets I. 2024. Neuropeptide signaling network of Caenorhabditis elegans: from structure to behavior. GENETICS. 228(3):iyae141. doi:10.1093/genetics/iyae141.

Xu K, Zheng X, Sehgal A. 2008. Regulation of Feeding and Metabolism by Neuronal and Peripheral Clocks in Drosophila. Cell Metab. 8(4):289–300. doi:10.1016/j.cmet.2008.09.006.

Yapici N, Zimmer M, Domingos AI. 2014. Cellular and molecular basis of decision‐making. Embo Rep. 15(10):1023–1035. doi:10.15252/embr.201438993.
